# Development of a rapamycin-inducible protein-knockdown system in the unicellular red alga *Cyanidioschyzon merolae*

**DOI:** 10.1093/plphys/kiae316

**Published:** 2024-06-04

**Authors:** Takayuki Fujiwara, Shunsuke Hirooka, Shota Yamashita, Fumi Yagisawa, Shin-ya Miyagishima

**Affiliations:** Department of Gene Function and Phenomics, National Institute of Genetics, Shizuoka 411-8540, Japan; Department of Genetics, Graduate University for Advanced Studies (SOKENDAI), Shizuoka 411-8540, Japan; Department of Gene Function and Phenomics, National Institute of Genetics, Shizuoka 411-8540, Japan; Department of Gene Function and Phenomics, National Institute of Genetics, Shizuoka 411-8540, Japan; Research Facility Center, University of the Ryukyus, Okinawa 903-0213, Japan; Department of Gene Function and Phenomics, National Institute of Genetics, Shizuoka 411-8540, Japan; Department of Genetics, Graduate University for Advanced Studies (SOKENDAI), Shizuoka 411-8540, Japan

## Abstract

An inducible protein-knockdown system is highly effective for investigating the functions of proteins and mechanisms essential for the survival and growth of organisms. However, this technique is not available in photosynthetic eukaryotes. The unicellular red alga *Cyanidioschyzon merolae* possesses a very simple cellular and genomic architecture and is genetically tractable but lacks RNA interference machinery. In this study, we developed a protein-knockdown system in this alga. The constitutive system utilizes the destabilizing activity of the FK506-binding protein 12 (FKBP12)-rapamycin-binding (FRB) domain of human target of rapamycin kinase or its derivatives to knock down target proteins. In the inducible system, rapamycin treatment induces the heterodimerization of the human FRB domain fused to the target proteins with the human FKBP fused to S-phase kinase-associated protein 1 or Cullin 1, subunits of the SCF E3 ubiquitin ligase. This results in the rapid degradation of the target proteins through the ubiquitin-proteasome pathway. With this system, we successfully degraded endogenous essential proteins such as the chloroplast division protein dynamin-related protein 5B and E2 transcription factor, a regulator of the G1/S transition, within 2 to 3 h after rapamycin administration, enabling the assessment of resulting phenotypes. This rapamycin-inducible protein-knockdown system contributes to the functional analysis of genes whose disruption leads to lethality.

## Teaser

A rapamycin-inducible protein-knockdown system developed in the unicellular red alga *Cyanidioschyzon merolae* enables functional analysis of essential genes.

## Introduction

Photosynthesis converts solar energy into chemical energy and produces various organic compounds, which represent a primary pathway through which energy enters ecosystems. Thus, the mechanisms of cellular growth, metabolism, and responses to environmental changes in photosynthetic organisms that thrive across terrestrial, freshwater, and marine environments have been the subject of extensive study.

Among several unicellular algae in which various experimental tools, including those for genetic manipulation procedures, have been developed, the unicellular red alga *Cyanidioschyzon merolae* and its experimental platform possess the following features (Miyagishima and Tanaka 2021). This organism exhibits an exceptionally simple intracellular organization, featuring a single nucleus, mitochondrion, chloroplast, Golgi apparatus, peroxisome, approximately 4 vacuoles, and a single layer of endoplasmic reticulum. The genomic content is also very simple, with the nuclear genome encoding a mere 4,775 proteins (Matsuzaki et al. 2004). Its cell cycle progression can be precisely synchronized with the diel cycle, providing substantial advantages for diverse omic analyses and investigations into organelle relationships (Miyagishima and Tanaka 2021). Moreover, because this alga lacks a rigid cell wall, the cellular contents can be easily extracted (Miyagishima and Tanaka 2021). We previously developed highly efficient procedures for editing multiple chromosomal loci through homologous recombination (Ohnuma et al. 2008; Imamura et al 2009; Fujiwara, Ohnuma, et al. 2013; Fujiwara et al. 2017; Takemura et al. 2018). Additionally, CRISPR-CAS9 technology has also been applied to *C. merolae* (Tanaka et al. 2021), and procedures for conditionally inducing the expression of transgenes and endogenous genes have been established (Sumiya et al. 2014; Fujiwara et al. 2015).

Initially, *C. merolae* has been used to study organelle proliferation (Miyagishima et al. 2003; Nishida et al. 2003; Yoshida et al. 2010), cell cycle progression (Kobayashi et al. 2011; Fujiwara, Tanaka, et al. 2013; Miyagishima et al. 2014; Yagisawa et al. 2020), and the metabolism supporting these processes (Imamura et al. 2009; Miyagishima et al. 2019). Furthermore, this organism has recently been applied to various research areas, including the characterization of the photosynthetic apparatus (Krupnik et al. 2013; Nikolova et al. 2017; Abram et al. 2020), epigenetics (Mikulski et al. 2017; Hisanaga et al. 2023), RNA processing (Stark et al. 2015; Schärfen et al. 2022), and industrial applications (Pancha et al. 2021; Seger et al. 2023; Villegas-Valencia et al. 2023). Recently, in the related genera *Galdieria*, which are facultative mixotrophs (i.e. they can grow either photoautotrophically, heterotrophically, or mixotrophically) in contrast to the obligately photoautotrophic *C. merolae*, procedures for genetic manipulation and induction of sexual reproduction have been developed (Hirooka et al. 2022).

The simplicity of *C. merolae*'s (and its relatives) cellular architecture and genome content should facilitate the characterization of mechanisms that are essential and common to photosynthetic eukaryotes. However, inactivating or modifying such essential mechanisms through gene manipulation often results in lethality. Therefore, procedures for conditional inactivation of a gene or protein are desired in such cases.

In several organisms, RNA interference (RNAi) is frequently employed (Fire et al. 1998; Elbashir et al. 2001). In some cases, it is also applicable for examining the function of essential genes by transient application of RNAi (AN et al. 2003; Huppi et al. 2005; Bjork et al. 2010; Andrieu et al. 2012). However, in several organisms, such as *C. merolae*, *Galdieria*, and the green algae *Ostreococcus*, RNAi does not work due to the absence of proteins involved in the RNAi mechanism, such as Argonaute and Dicer (Casas-Mollano et al. 2008; Cerutti et al. 2011). Independent losses of RNAi have also been observed in various lineages of fungi (Billmyre et al. 2013) and kinetoplastids (Matveyev et al. 2017). Beyond RNAi, recent studies in yeasts and mammalian cell lines have begun to use alternative approaches to investigate the functions of essential proteins. These approaches are known as protein-knockdown or degron systems, in which a target protein is directly degraded upon the addition of a certain chemical to the culture (Kanemaki 2022). In comparison with transient RNAi, there are several advantages to this approach, including a very rapid onset of effects (within minutes to a few hours) and the ability to remove proteins that are already present within the cell, which is not feasible with transient RNAi (Kanemaki 2022). Furthermore, protein-knockdown systems can also prove that the inability to generate a loss-of-function mutation is solely attributable to the protein loss being actually lethal, rather than the combination of the protein loss and stress on the cells caused by the transformation process.

To facilitate studies on genes and mechanisms essential for survival and growth in *C. merolae*, in this study, we have developed both constitutive and inducible protein-knockdown systems in this alga. Firstly, for the former, we utilized the FKBP-rapamycin-binding (FRB) domain of human target of rapamycin (TOR) kinase. A target protein fused with FRB (Stankunas et al. 2003) via genetic modification was destabilized, leading to a constitutive decrease in its protein level. Furthermore, the latter was designed to further degrade an FRB-fused protein, whose level had been reduced by FRB fusion, upon treatment with rapamycin. This was achieved using the FRB domain and the human FK506-binding protein 12 (FKBP), along with rapamycin, which induces their heterodimerization (Chen et al. 1995), in addition to the ubiquitin-proteasome pathway. The inducible protein-knockdown system utilizing a dimerization-inducing chemical developed in *C. merolae* in this study is currently the sole technology among photosynthetic eukaryotes and would facilitate functional analyses of essential proteins in photosynthetic eukaryotes.

## Results

### Development of a constitutive protein-knockdown system using the FRB domain as a destabilizing domain tag

**Figure 1.**
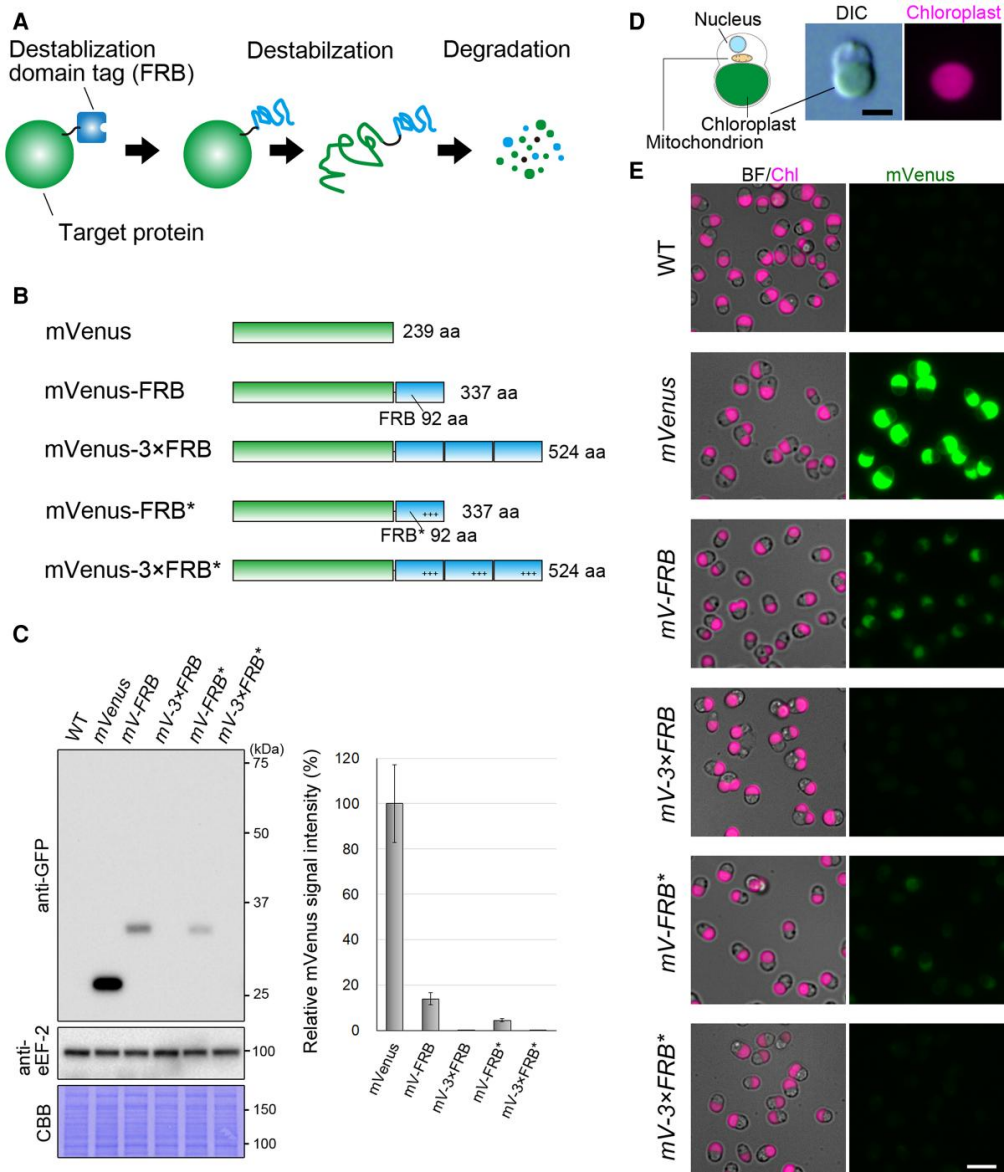
A constitutive protein knockdown utilizing the human FRB domain and its derivative in *C. merolae*. **A)** A schematic illustration of the degradation process of an FRB-conjugated target protein. The presence of FRB leads to the destabilization of the target protein, ultimately resulting in the degradation of both the FRB and the target protein. **B)** The structures of mVenus (the target protein here) and the 4 types of human FRB-fused mVenus expressed in *C. merolae*. FRB, 3×FRB, FRB*, and 3×FRB* tags were attached to the carboxy-terminus of mVenus. FRB* contains 3 mutations (K72P, T75L, and W78F), denoted with + in the illustration, which has been reported to exhibit a higher destabilizing effect than the WT FRB (Stankunas et al. 2003). Although not indicated in the illustration, there is a 6×G peptide linker between mVenus and any FRB tag. **C)** Immunoblotting comparing the efficiency of the 4 different types of FRB tags in reducing mVenus level in the cells. The left panel shows the immunoblot using the anti-GFP antibody, which reacts with mVenus, to compare the levels of mVenus in the cells expressing mVenus alone or mVenus conjugated with the 4 types of FRB tags. The WT cell was used as a negative control. The immunoblot using the eukaryotic translation elongation factor 2 (eEF-2) antibody and the Coomassie brilliant blue (CBB)-stained PVDF membrane are also shown as loading controls. The predicted molecular weights based on the amino acid sequence of mVenus, mVenus-FRB, mVenus-3×FRB, mVenus-FRB*, and mVenus-3×FRB* are 26, 37, 58, 37, and 58 kDa, respectively. The right panel shows a bar graph quantifying the protein levels. The signal intensity of mVenus alone was set as 100%. The values represent the mean of technical triplicate of the immunoblotting results, and the bars indicate the Sd. Note that both 3×FRB-fused and 3×FRB*-fused mVenus proteins were undetectable in the immunoblotting. **D)** An illustration, as well as the differential interference contrast (DIC) and fluorescent micrographs of the *C. merolae* cell in the G1 phase. The cell possesses a single chloroplast and mitochondrion. The chloroplast emits red fluorescence. The scale bar represents 2 *µ*m in all images in **D)**. **E)** Fluorescent microscopic images of the cells expressing mVenus or that conjugated with the 4 different kinds of FRB tag. WT served as a negative control. The images in the left and right column represent autofluorescence from chloroplasts (Chl) and mVenus fluorescence, respectively. For the images of Chl, those of the bright field (BF) were merged. The scale bar represents 5 *µ*m in all images in **E)**.

In this study, we tried to develop both constitutive and conditional protein-knockdown systems in *C. merolae*. First, in order to develop a constitutive knockdown system, we focused on the FRB domain of TOR kinase responsible for sensing intracellular metabolic status and regulating cell growth and proliferation (Chung et al. 1992). It has been shown that when this domain is conjugated to a protein, it facilitates its degradation. While the exact mechanism is not yet fully understood, the FRB domain is inherently unstable, likely leading to the degradation of both itself and the conjugated protein through proteasomal pathways (Fig. 1A; Stankunas et al. 2003). Additionally, a variant of the FRB domain, known as FRB*, which possesses 3 nonsynonymous mutations, has been reported to exhibit a higher destabilizing effect than the wild-type (WT) FRB (Stankunas et al. 2003). To test whether FRB and FRB* are applicable for constitutive protein knockdown in *C. merolae*, we generated 5 types of *C. merolae* transformants expressing the fluorescent protein mVenus alone or each with one of 4 kinds of FRB variants (a single or triple human FRB or FRB*) at the C-terminus (Fig. 1B). These proteins were designed to be constitutively expressed from a chromosomal neutral site (Fujiwara et al. 2015) by the *EF-Tu* promoter. They are referred to as the *mVenus*, *mV-FRB*, *mV-3×FRB*, mV-FRB*, and mV-3×FRB* strains. Using these strains, we assessed the efficiency of the 4 types of FRB variants in mediating mVenus protein degradation in *C. merolae* cells.

The immunoblotting analysis of the total cellular proteins showed that mVenus-FRB and mVenus-FRB* levels were reduced to 16% and 5%, respectively, compared with mVenus alone (Fig. 1C). In addition, the levels of mVenus-3×FRB and mVenus-3×FRB* were reduced to undetectable levels (Fig. 1C). Fluorescence microscopy further confirmed these results, showing that mVenus fluorescence in the *mV-FRB* and mV-FRB* strains was substantially reduced in comparison with the *mVenus* strain (Fig. 1, D and E). The mVenus fluorescence in the *mV-3×FRB* and mVs-3×FRB* strains was almost undetectable (Fig. 1E). These results indicate that an enhanced degradation efficiency of the FRB* tag over the FRB tag and that the triple-tag exhibits higher efficiency than the single tag in *C. merolae*, consistent with the results in other organisms (Stankunas et al. 2003; Schwartz et al. 2007).

### Application of FRB tags for constitutive knockdown of the endogenous RB protein

**Figure 2.**
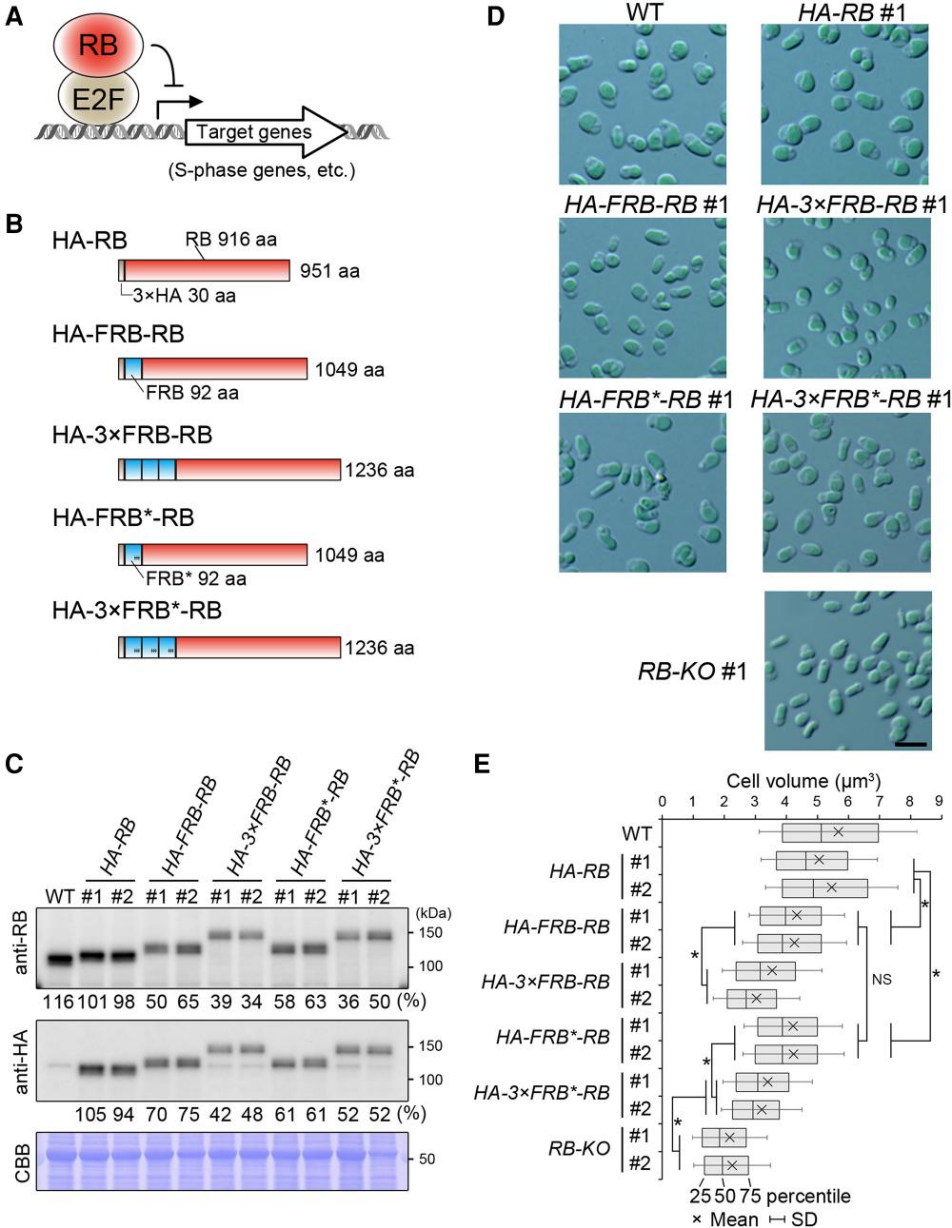
Application of an FRB-tag-mediated knockdown for RB protein. **A)** A schematic illustration of the function of RB as a negative regulator of G1/S transition. RB directly binds E2F and inhibits E2F-DP heterodimer to transcribe S-phase genes. The arrow line indicates positive regulation of the transcription of S-phase genes by E2F, whereas the T-shaped line indicates negative regulation by RB. CDK-cyclin accumulates in accordance with cellular growth during the G1 phase, phosphorylating RB, which results in RB inactivation and allows E2F-DP to transcribe S-phase genes. **B)** The structures of HA-RB and the 4 types of FRB-inserted HA-RB variants expressed in *C. merolae*. FRB, 3×FRB, FRB*, and 3×FRB* were inserted between HA and the amino terminus of RB. FRB* contains 3 mutations (K72P, T75L, and W78F), denoted with a + in the illustration. Although not indicated in the illustration, a GGGGS peptide linker was inserted between 3× HA and RB in the HA-RB protein. Additionally, a 6×G peptide linker was inserted between each FRB tag and RB. **C)** Immunoblotting comparing the efficiency of the 4 different types of FRB tags in reducing RB level in the cells. RB and HA-RB were detected using the anti-RB and an anti-HA antibody, respectively. For each strain other than WT, the results of 2 independent transformants (#1 and #2) are shown. The predicted molecular weights based on the amino acid sequence of RB, HA-RB, HA-FRB-RB, HA-3×FRB-RB, HA-FRB*-RB, and HA-3×FRB*-RB are 101, 105, 115, 136, 115, and 136 kDa, respectively. The values below the bands indicate the relative signal intensities of RB, HA-RB, and HA-FRB-RB proteins. The average signal intensity of 2 independent clones (#1 and #2) of HA-RB was set to 100%. The CBB-stained PVDF membrane is also shown as a loading control. **D)** DIC images of WT, *HA-RB*, and the 3 kinds of *HA-FRB-RB* strains. The results for Clone #1 are presented for each strain. Images of Clone #1 and Clone #2 for each strain are provided in Supplementary Fig. S1. The scale bar represents 5 *µ*m in all images in **D)**. **E)** A box plot comparing the cell volume of WT, *HA-RB*, the 3 types of *HA-FRB-RB*, and *RB-KO* strains. The results for 2 independent clones (#1 and #2) are shown for each transformant. The box extends from the first to the third quartile, with the vertical line representing the median, the cross indicating the mean, and the error bar indicating the Sd. NS, not significant; *P < 0.01 (Student’s *t*-test; pairwise comparisons between the 2 clones). The histograms representing the distribution of cell volume in respective strains are shown in Supplementary Fig. S1.

To assess whether protein knockdown using the FRB tag is applicable for the analysis of endogenous proteins, we conducted a knockdown of the retinoblastoma (RB) protein (CMT038C) as a case study in *C. merolae*. RB is an inhibitor of the G1/S transition of the cell cycle in red and green algae (Umen and Goodenough 2001; Miyagishima et al. 2014), as it is in other eukaryotes (Knudsen et al. 1998; De Veylder et al. 2002; Chen et al. 2009). RB inhibits the E2 transcription factor (E2F)-dimerization partner (DP) heterodimer from functioning as a transcription factor for the S-phase genes by directly binding to E2F (Fig. 2A). During the G1 phase, G1 cyclin accumulates as a result of cell growth, and the CDK-G1 cyclin complex phosphorylates RB. This phosphorylation inactivates RB, thus activating the transcription of S-phase genes by E2F-DP (Buchkovich et al. 1989; Burke et al. 2010; Kent and Leone 2019).

In our previous study, we showed that *C. merolae RB* knockouts exhibited a small cell phenotype (Miyagishima et al. 2014), similar to *RB* mutants in the green alga *Chlamydomonas reinhardtii* (Umen and Goodenough 2001). This is probably because, in RB knockouts, the G1/S transition occurs even without sufficient cell growth and accumulation of G1 cyclin.

To examine the efficiency of FRB tags on reducing the endogenous RB protein level and their effects on the cell size, we generated *C. merolae* transformants that express HA-RB and 4 types of HA-FRB-RB from the chromosomal *RB* locus. These are referred as the *HA-RB*, *HA-FRB-RB*, *HA-3×FRB-RB*, HA-FRB*-RB, and HA-3×FRB*-RB strains (Fig. 2B). The 3×HA tag (for immunological detection of the protein) and respective FRB tags were conjugated to the N-terminus of RB (Fig. 2B). For comparison, we also generated an RB knockout (*RB-KO*) strain. Then, the WT, the 5 types of transformed strains, and *RB-KO* strains were cultured asynchronously for 3 d and respective log-phase cultures were examined.

**Figure 3.**
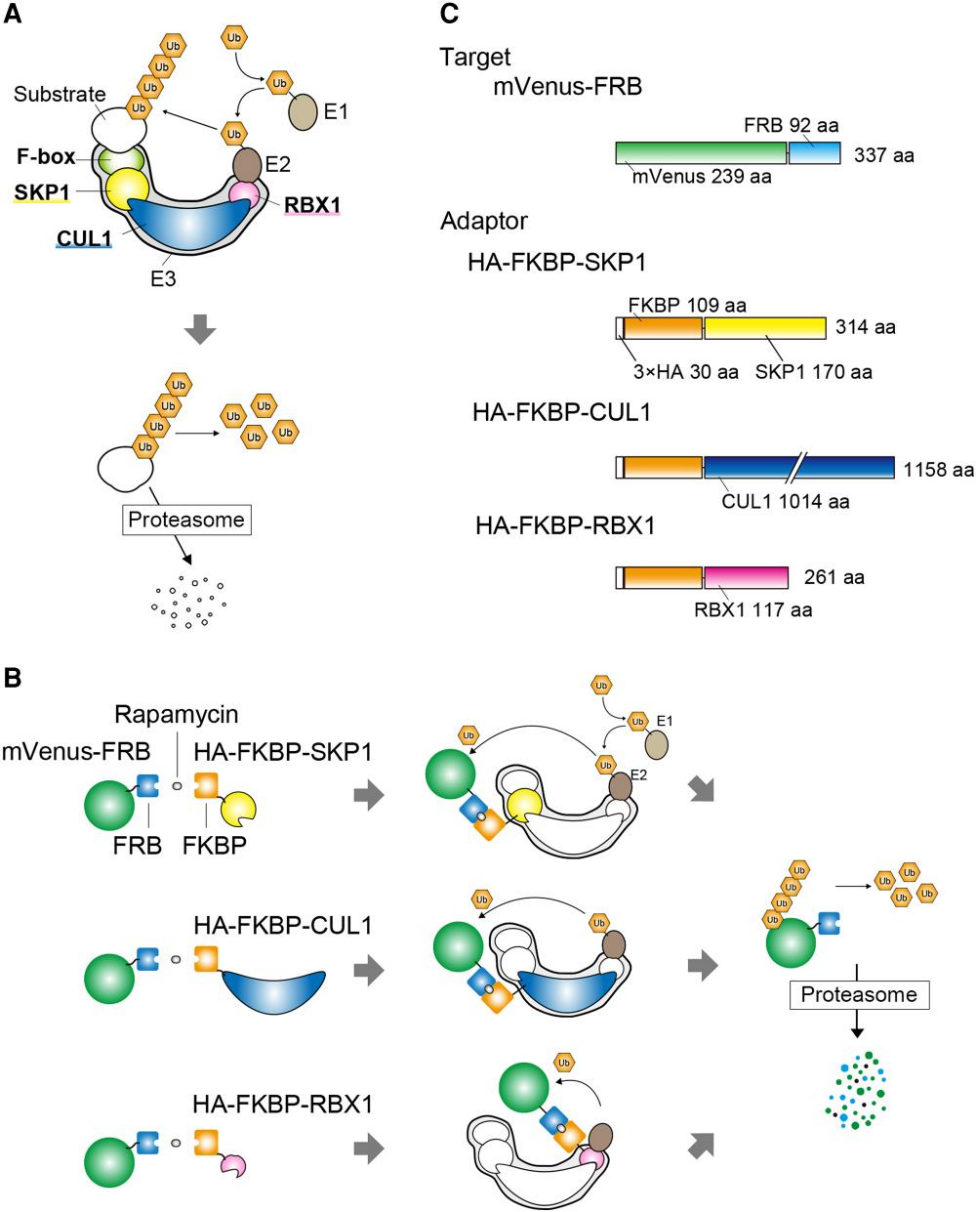
Diagram of a rapamycin-inducible protein-knockdown system. **A)** The flow of ubiquitination of a substrate protein through the SCF E3 ligase complex, which results in degradation of the substrate protein in proteasome. An E1 ubiquitin-activating enzyme initiates ubiquitination by activating and transferring a ubiquitin to an E2 ubiquitin-conjugating enzyme. The E2 enzyme then ubiquitinates a substrate protein captured by the SCF complex. The SCF complex consists of a F-box protein, SKP1, CUL1, and RBX1. The F-box proteins determine the substrate specificity for the SCF complex. SKP1 mediates the interaction between F-box proteins and CUL1, which serves as a primary scaffold protein. RBX1 connects CUL1 to the E2 ubiquitin-conjugating enzyme. **B)** The experimental design of the rapamycin-inducible protein-knockdown system. The SCF E3-ligase complex was employed for ubiquitination of a target protein. The rapamycin-mediated heterodimerization of FRB domain (of TOR kinase) and FKBP protein was utilized to facilitate the incorporation of the target protein (mVenus, in this case) into the SCF complex. By fusing the human FRB domain to a target protein and expressing an SCF complex component fused with the human FKBP, followed by the addition of rapamycin, the target protein is expected to be captured by the SCF complex and degraded via the ubiquitin-proteasome pathway. In this study, we evaluated the efficiency of fusing FKBP to SKP1, CUL1, or RBX1 as SCF components for the degradation of mVenus-FRB upon the addition of rapamycin. **C)** The structures of mVenus-FRB, HA-FKBP-SKP1, HA-FKBP-CUL1, and HA-FKBP-RBX1 expressed in *C. merolae*.

Immunoblotting with the anti-RB (Miyagishima et al. 2014) and anti-HA antibodies confirmed the successful establishment of 2 independent clones for each transformant (Fig. 2C). Regarding the RB protein level, the immunoblotting showed the following: (i) in either the case of FRB or FRB*, the 3× tagging exhibited higher efficiency than a single tagging for reducing the RB level; (ii) but the efficiency is not obviously different between FRB or FRB* (Fig. 2C) in contrast to the results of mVenus (Fig. 1); (3) even in the most efficient case, while almost all mVenus was erased (Fig. 1), the FRB tagging only reduced the RB protein level to approximately 40% of the WT (Fig. 2C). However, microscopic observation and quantitative examination by a Coulter counter (Beckman) showed a reduction in cellular size, apparently in a dose-dependent manner, associated with the reduction of RB protein levels (Fig. 2, D and E; Supplementary Fig. S1). *RB-KO* cells were even smaller than the 3×FRB- and 3×FRB*-RB, indicating that approximately 40% to 50% of RB, which were fused with 3×FRB- and 3×FRB*, were still physiologically functional but not sufficient to maintain normal cell size (Fig. 2, C and E). These results suggest that not only the levels of G1 cyclin-CDK but also the levels of RB are likely crucial for cell size-dependent G1/S transition.

The reason for the lower efficiency of the FRB tag for the reduction of RB level (Fig. 2) when compared with that on mVenus (Fig. 1) is still unclear. However, the binding of several other proteins such as E2F and other RB-binding proteins (Shen 2002; Shevchenko 2010; Ishak et al. 2016) likely, to a certain extent, protects FRB-tagged RB from degradation.

### Development of a rapamycin-inducible rapid protein degradation method in *C. merolae*

We demonstrated that fusion with the FRB tag leads to a reduction in the targeted protein levels (Figs. 1 and 2). However, depending on the type of protein, a reduction in protein levels due to fusion with FRB alone may not always result in readily observable phenotypes. To develop a conditional protein-knockdown system in *C. merolae*, we focused on the rapamycin-mediated heterodimerization between the FRB domain of TOR and the FKBP protein (Chen et al. 1995). We aimed to artificially integrate this rapamycin-mediated dimerization into the ubiquitin-proteasome pathway, which plays a crucial role in the elimination of damaged, misfolded, or unnecessary proteins (Langmead and Salzberg 2012; Chhangani et al. 2013). Protein ubiquitination in this system involves a 3-step process: ubiquitin activation, mediated by an E1 enzyme, ubiquitin conjugation, which involves E2 enzymes, and ubiquitin ligation, facilitated by E3 enzymes. After ubiquitination, the ubiquitinated proteins are degraded by proteasomes (Buetow and Huang 2016) (Fig. 3A). One of the most extensively studied E3 ligases is the SCF (Skp, Cullin, F-box containing) complex, which exhibits wide conservation among eukaryotes. The SCF complex consists of an F-box protein, S-phase kinase-associated protein 1 (SKP1), Cullin 1 (CUL1), and Ring-Box 1 (RBX1), each of which functions in substrate binding, acts as an adapter for F-box proteins, provides structural scaffolding, and binds to E2 enzymes, respectively (Fig. 3A). By utilizing this system, our idea was that the human FRB-fused target protein and the human FKBP-fused E3 ligase would heterodimerize specifically in the presence of rapamycin, leading to the degradation of the target protein (Fig. 3B). Thus, in this system, an FRB fusion to a target protein, due to the destabilizing effect of FRB, constitutively reduces the level of the target protein to a certain extent (Figs. 1 and 2). The remaining FRB-fused target protein will further undergo degradation upon rapamycin treatment (Fig. 3). It should be noted that a previous study showed that *C. merolae* is not susceptible to rapamycin due to the insensitivity of its FKBP, unlike FKBP in other organisms (Imamura et al. 2013).

It should be noted that, during this study, a similar but different method for a rapamycin-mediated protein-knockdown system was developed in mammalian cultured cell lines. In this system, FRB is fused to the RING domain of LNX1, which functions as an E3 ligase but is distinct from the SCF complex, while FKBP is fused to a nanobody, which is a single-domain antibody designed to interact with the target protein (Deng et al. 2020).

Among the components of the SCF complex, the *C. merolae* genome (Matsuzaki et al. 2004) encodes 5 F-box proteins (Kobayashi et al. 2011; CMC028C, CMI121C, CMJ108C, CMM061C, and CMM138C), a single SKP1 (CMP118C), CUL1 (CMT046C), and RBX1 (CMQ353C) protein. The F-box protein specifically binds to a certain group of target proteins for their degradation, and thus, constitutive or overexpression of an F-box protein probably leads to excessive degradation of those endogenous proteins. In addition, it is still unknown which F-box protein is the most abundant or constitutively expressed in *C. merolae*. Because of these reasons, we tested the effects of human FKBP-fused SKP1, CUL1, or RBX on the degradation of a human FRB-fused target protein in a rapamycin-dependent manner. To this end, we generated *C. merolae* transformants that constitutively express 3×HA-FKBP-SKP1, 3×HA-FKBP-CUL1, or 3×HA-FKBP-RBX (by the *APCC* promoter) along with mVenus-FRB (by the *EF-Tu* promoter) from a chromosomal neutral site. These are referred as the *mV^RD-SKP1^*, *mV^RD-CUL1^*, and *mV^RD-RBX1^* strains (Fig. 3C).

**Figure 4.**
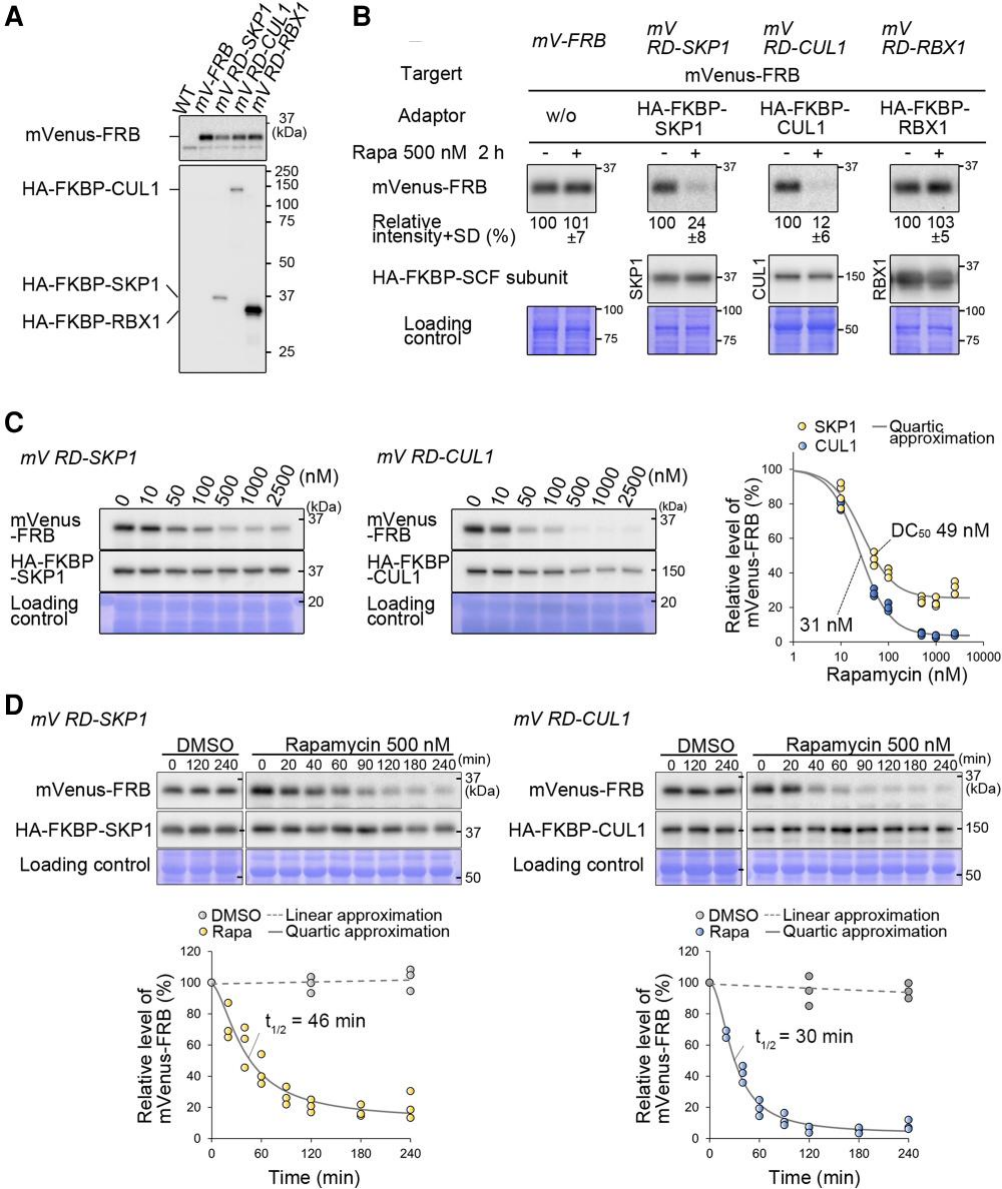
Evaluation of the rapamycin-inducible protein-knockdown system in *C. merolae*. **A)** Immunoblotting using the anti-HA antibody confirmed the expression of HA-FKBP-SKP1, HA-FKBP-CUL1, and HA-FKBP-RBX1 in the *mV^RD-SKP1^*, *mV^RD-CUL1^*, and *mV^RD-RBX1^* strains, respectively, where mVenus-FRB is also expressed. The predicted molecular weights based on the amino acid sequence of mVenus-FRB, HA-FKBP-SKP1, HA-FKBP-CUL1, and HA-FKBP-RBX1 are 37, 37, 127, and 29 kDa, respectively. WT and *mV-FRB* strains were used as negative controls. **B)** Immunoblotting comparing the efficiency of rapamycin-induced degradation of mVenus-FRB in the *mV^RD-SKP1^*, *mV^RD-CUL1^*, and *mV^RD-RBX1^* strains. For each culture, 500 nm rapamycin or DMSO (a control) was added and the cells were harvested 2 h after the addition. The *mV-FRB* was used as a negative control. mVenus-FRB protein was detected with the anti-GFP antibody. The CBB-stained PVDF membrane is also shown as a loading control. The values below the bands indicate the relative signal intensities of the mVenus-FRB protein. The average and Sd of immunoblotting of 3 independent transgenic clones are indicated for each strain. The signal intensity in each strain in the absence of rapamycin was set to 100%. **C)** Degradation efficiency of mVenus-FRB at different concentrations of rapamycin. Immunoblotting showing the relative level of mVenus-FRB protein in the *mV^RD-SKP1^* and *mV^RD-CUL1^* culture 2 h after the addition of 0, 10, 50, 100, 500, 1,000, and 2,500 nm rapamycin. The accompanying graph shows the change in mVenus-FRB protein level obtained from biological triplicates. The signal intensity in each strain in the absence of rapamycin was set to 100%. The degradation concentrations (DC50) in *mV^RD-SKP1^* and *mV^RD-CUL1^*, as shown in the graph, were calculated using the quartic approximation equations in Microsoft Excel. **D)** Degradation kinetics of mVenus-FRB protein. Immunoblotting showing the time course of the changes in the relative level of mVenus-FRB protein in *mV^RD-SKP1^* and *mV^RD-CUL1^* culture after the addition of 500 nm rapamycin. The accompanying graph illustrates the changes in mVenus-FRB protein levels, quantified from biological triplicate immunoblotting. The signal intensity just before the rapamycin addition in each strain was set to 100%. The half-lives (*t*_1/2_) of mVenus-FRB in the *mV^RD-SKP1^* and *mV^RD-CUL1^* strains, as shown in the graph, were calculated using the quartic approximation equations in Microsoft Excel.

Immunoblotting using anti-GFP and anti-HA antibodies confirmed the successful generation of *mV^RD-SKP1^*, *mV^RD-CUL1^*, and *mV^RD-RBX1^* strains (Fig. 4A). To examine whether rapamycin-mediated degradation of mVenus-FRB occurs in these strains, the *mV-FRB* (as a negative control), *mV^RD-SKP1^*, *mV^RD-CUL1^*, and *mV^RD-RBX1^* log-phase cultures were treated with 500 nm rapamycin for 2 h (Fig. 4B). Immunoblotting showed an effective decrease in the mVenus-FRB protein level in the *mV^RD-SKP1^ and mV^RD-CUL1^* strains, but not in the *mV^RD-RBX1^* strain. The reason for the no obvious degradation of mVenus-FRB in the *mV^RD-RBX1^* strain is unclear but HA-FKBP-RBX1 likely failed to be integrated into the SCF complex, or the positioning of HA-FKBP-RBX1-bound mVenus-FRB within the complex was inappropriate for ubiquitination.

Then, we assessed the efficiency of mVenus-FRB degradation in the *mV^RD-SKP1^* and *mV^RD-CUL1^* strains in the presence of various concentrations of rapamycin (Fig. 4C). In the *mV^RD-SKP1^* and the *mV^RD-CUL1^* strains, the degradation concentration 50 (DC50) for mVenus was 49 and 31 nm, and the mVenus protein level reached a minimum at ∼25% and ∼5% with 500 nm or higher concentrations of rapamycin, respectively. Regarding the kinetics of the mVenus degradation, the half-lives (t1/2) of mVenus proteins was 46 or 30 min in the *mV^RD-SKP1^* or *mV^RD-CUL1^* strain, respectively, and the protein level reached a minimum 2 h after the rapamycin addition in both strains (Fig. 4D). In summary, among the 3 strains we have prepared, *mV^RD-CUL1^* in which FKBP is fused to CUL1 is the most effective to degrade mVenus-FRB.

Then, we examined the duration of rapamycin-induced degradation of mVenus-FRB in the *mV^RD-CUL1^* strain. The mVenus-FRB level initially decreased until 4 h after the rapamycin dose (500 nm) but started to recover afterward while HA-FKBP-CUL1 protein level kept constant (Supplementary Fig. S2). We suspected that the recovery of mVenus-FRB level might be due to rapamycin inactivation in the acidic culture medium (pH 2.3) or unknown metabolic processes in the *C. merolae* cell. Supporting this assumption, the recovery of mVenus-FRB levels was temporarily impeded by the second rapamycin dose 6 h after the first dose and the third dose 10 h after the first dose (each dose was 500 nm) (Supplementary Fig. S2). Thus, periodical addition of rapamycin can continue to promote the degradation of the target protein in this system developed in *C. merolae*.

As described above, the *C. merolae* WT is not susceptible to rapamycin due to the rapamycin-insensitive nature of the *C. merolae* FKBP protein. However, rapamycin might exert some inhibitory effect on the TOR kinase through the human FKBP, which is heterologously expressed in our rapamycin-inducible protein degradation system. To assess the potential side effect of rapamycin on the cells expressing FKBP-CUL1, we compared cellular growth and transcriptome profiles (RNA-seq data) of the *mV^RD-SKP1^* strain in the presence and absence of rapamycin (Supplementary Fig. S3). The same experiments were also conducted for the WT for comparison (Supplementary Fig. S3).

Regarding the cellular growth rate, 500 nm rapamycin dose did not affect the growth rate of the *mV^RD-SKP1^* culture as well as the WT culture for 14 d (Supplementary Fig. S3A). Regarding the transcriptome of the culture 2 h after the 500 nm rapamycin dose, no differentially expressed gene (DEG; false discovery rate (FDR) < 0.01, log2 fold-change > 1 or <−1) was detected between the culture in the presence and absence of rapamycin in the WT (Supplementary Fig. S3B). In contrast, in the *mV^RD-SKP1^* culture, the rapamycin treatment resulted in upregulation of 30 genes (0.6%) and downregulation of 16 genes (0.3%) among the 4,775 nucleus-encoding genes (Supplementary Fig. S3B and Table S1). The upregulated genes included those associated with the nitrogen-deficient response (Imamura et al. 2009, 2015; Supplementary Table S1) while the downregulated genes included those encoding proteins for translation in the chloroplast and chloroplast molecular chaperones (Supplementary Fig. S3B). However, the magnitudes of the changes in the DEGs detected in the *mV^RD-SKP1^* strain were relatively small and the changes likely have little impact on cellular activity.

While the side effect of rapamycin was faint in *C. merolae* expressing the human FKBP, in an effort to eliminate it entirely, we explored an alternative approach and focused on a rapamycin analog, AP21967 (A/C heterodimerize), in combination with the FRB variant FRB^T75L^, which corresponds to human TOR1^T2098L^. AP21967 has the ability to heterodimerize FKBP and FRB^T75L^ but not the WT FRB domain of TOR and thus has no effect on endogenous TOR1 (Bayle et al. 2006). To test whether this combination of AP21967 and TOR1^T2098L^ is applicable to *C. merolae* protein knockdown, we generated the *mV^TL-SKP1^* strain, which expresses mVenus-FRB^T75L^ and HA-FKBP-SKP1. The *mV^TL-SKP1^* culture was treated with 1 or 5 *μ*m AP21967 for 2, 4, or 6 h. However, in any case, mVenus-FRB^T75L^ level did not decrease (Supplementary Fig. S4). Thus, for further improvement of the system, other combinations of rapamycin analog and FRB variants need to be tested in *C. merolae*.

### Addition of the Stabilon tag counteracted the destabilizing activity of FRB on a target protein

Another concern in the rapamycin-induced protein-knockdown system is that the FRB tagging reduces the stability and thus the basal level of a target protein before addition of rapamycin. As observed above, FRB tagging reduced exogenous mVenus level to ∼20% and endogenous RB level to 70% to 75% compared with respective proteins without FRB tagging. The difference in the reduction of the protein level by the FRB tag between the mVenus and RB is likely due to the difference of original stability of mVenus, which is structurally very stable (Li et al. 1998), and RB.

To address this issue, we tested the use of the Stabilon tag. The Stabilon tag is the C-terminal 13 amino acids of the p54/Rpn10 ubiquitin receptor subunit of the 26S proteasome in *Drosophila melanogaster* and increases the stability of a tagged protein although the precise mechanism of the stabilization remains unclear (Rethi-Nagy et al. 2022). To test the effect of Stabilon tag in the rapamycin inducible protein-knockdown system, we generated the Stab-*mV^RD-CUL1^* and *mV-*Stab*^RD-CUL1^* strain, which expresses the N- or C-terminally Stabilon-tag-fused mVenus-FRB, respectively, and HA-FKBP-SKP1 (Supplementary Fig. S5). When the Stabilon tag was fused to mVenus, the reduction of basal mVenus level caused by the FRB tag before the rapamycin addition was partially mitigated, although not completely. This stabilizing effect was higher when the Stabilon tag was added at the C-terminus compared with when it was added at the N-terminus (Supplementary Fig. S5). To examine the effect of the Stabilon tag on rapamycin-induced degradation of mVenus, we then examined Stabilon tagged 2 h after the rapamycin addition. The results showed that while the rapamycin addition induced degradation of the Stabilon-tagged mVenus, the remaining mVenus level after the addition increased proportionally to the increase in the basal mVenus level caused by the Stabilon tag (Supplementary Fig. S5). Based on these results, we conclude that whether to utilize the Stabilon tag should be determined based on the property of a target protein.

### Rapamycin-induced endogenous DRP5B knockdown successfully inhibited the chloroplast division

**Figure 5.**
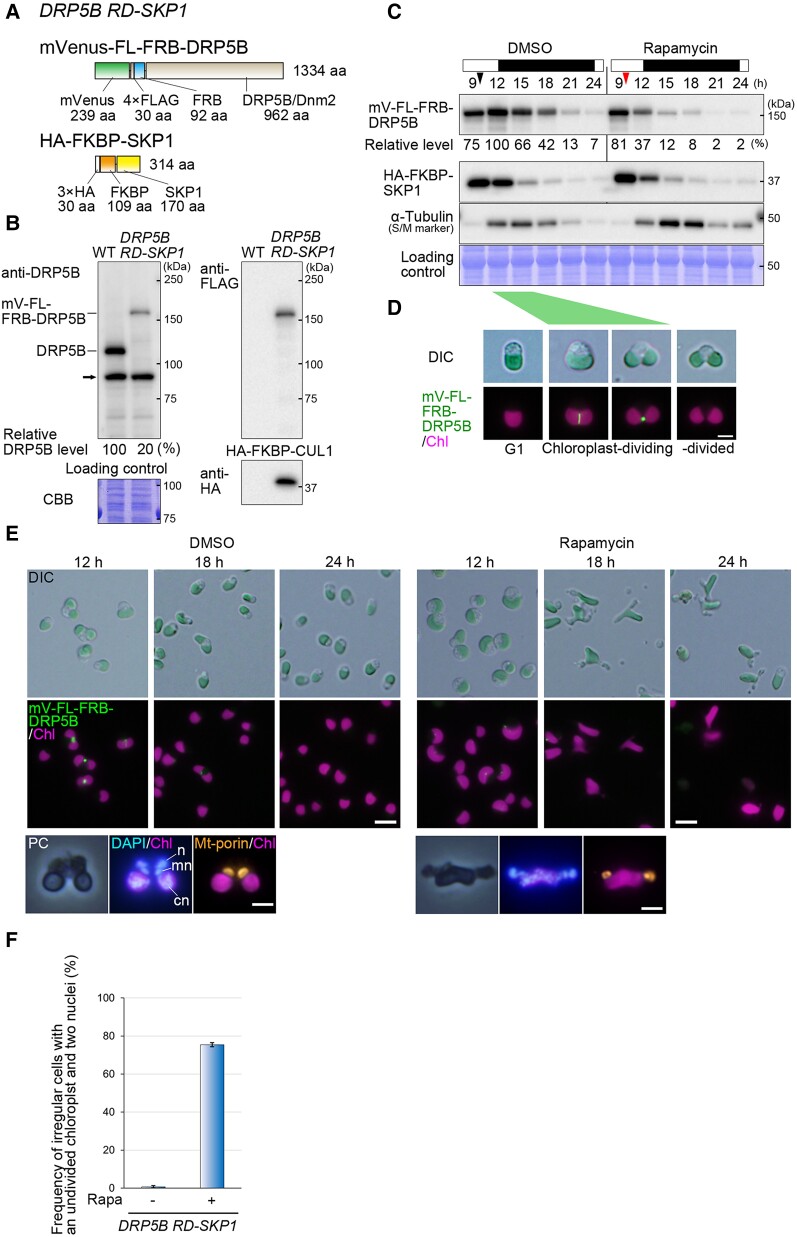
Application of the rapamycin-inducible knockdown system for the chloroplast division protein DRP5B. **A)** The structures of mVenus-FL-FRB-DRP5B and HA-FKBP-SKP1 expressed in the *DRP5B^RD-SKP1^*. mVenus, 4×FLAG (FL), and FRB were fused to the amino-terminus of DRP5B for visualizing protein localization, immunological protein detection, and rapamycin-inducible interaction with HA-FKBP-SKP1, respectively. A GSGSG peptide linker was inserted between mVenus and FL. Additionally, a 6×G peptide linker was inserted between FRB and DRP5B. The structure of the HA-FKBP-SKP1 is shown in Fig. 3. **B)** Immunoblotting confirmed expression of mVenus-FL-FRB-DRP5B and HA-FKBP-SKP1 in the *DRP5B^RD-SKP1^* knock-in strain. An anti-DRP5B antibody detected DRP5B protein in WT, while in *DRP5B^RD-SKP1^* strain, only mVenus-FL-FRB-DRP5B protein was detected indicating that *mVenus-FL-FRB-DRP5B* was successfully integrated into the chromosomal *DRP5B* locus. The arrow indicates a nonspecific band. Anti-FLAG and anti-HA antibodies detected mVenus-FL-FRB-DRP5B and HA-FKBP-SKP1, respectively, in the *DRP5B^RD-SKP1^* strain. The CBB-stained PVDF membrane is also shown as a loading control. The predicted molecular weights based on the amino acid sequence of DRP5B, mVenus-FL-FRB-DRP5B, and HA-FKBP-SKP1 are 106, 146, and 37 kDa, respectively. The values below the bands indicate the relative signal intensities of the DRP5B (DRP5B or mV-FL-FRB-DRP5B) protein. The signal intensity of DRP5B in WT was set to 100%. **C)** Rapamycin-induced degradation of mVenus-FL-FRB-DRP5B protein in the *DRP5B^RD-SKP1^* strain. Cell cycle progression and accompanying chloroplast division were synchronized under the LD cycle. The white and black bars above the immunoblot represent light and dark periods, respectively. Immunoblotting using the anti-FLAG antibody shows the change in the level of mVenus-FL-FRB-DRP5B protein during the LD cycle. DMSO, as a negative control, or 500 nm rapamycin was added at Hour 9 (indicated by the red arrowhead), which corresponds to 3 h prior to the peak of mVenus-FL-FRB-DRP5B protein level in the culture without rapamycin. The change in α-tubulin (detected by the anti-α-tubulin antibody; expressed specifically during S and M phases in *C. merolae*; Fujiwara, Tanaka, et al. 2013) is also shown. The CBB-stained PVDF membrane is shown as a loading control. The values below the bands indicate the relative signal intensities of the mV-FL-FRB-DRP5B protein. The signal intensity at Hour 12 in the absence of rapamycin was set to 100%. **D)** DIC and fluorescent microscopy of the *DRP5B^RD-SKP1^* cells. The images were aligned in the order of cell cycle progression (note that the images are not from an identical cell). The top panels represent the cell before, during, and after chloroplast division. The lower panels are merged images of the mVenus fluorescence of mVenus-FL-FRB-DRP5B and the autofluorescence from chloroplasts (Chl). As observed in WT cells (Miyagishima et al. 2003), mVenus-FL-FRB-DRP5B proteins localize at the chloroplast division site. As in WT cells, in *DRP5B^RD-SKP1^* cells, the chloroplast division synchronously occurs between Hours 12 and 15 under the LD. The green structures within the cell are chloroplasts. The scale bar represents 2 *µ*m in all images in **D)**. **E)** The chloroplast division defect caused by the degradation of mVenus-FL-FRB-DRP5B. DIC and fluorescent images of DMSO- or rapamycin-treated *DRP5B^RD-SKP1^* cells at Hours 12, 18, and 24 under LD are shown. In the DMSO-treated culture, cells with dividing chloroplast and the mVenus-FL-FRB-DRP5B signal at the division site accumulated at Hour 12, while most of the cells had completed chloroplast division at Hour 18. In contrast, in the rapamycin-treated culture, mVenus-FL-FRB-DRP5B was faintly detectable, and constriction of the chloroplast division site was not observed in any cells at Hour 12. At Hours 18 and 24, irregularly shaped cells with a single chloroplast accumulated. The scale bar represents 5 *µ*m in the upper and middle images in **E)**. The bottom panels show the phase-contrast (PC), DAPI-staining, and immunofluorescent images of the *DRP5B^RD-SKP1^* cells. The bottom-left image represents a typical telophase cell in which chloroplast division and following mitochondrial division and chromosome segregation have been completed. The bottom-right image represents an irregularly shaped cell in which chloroplast division has been blocked, while mitochondrial division and chromosome segregation have been completed. *n*, nuclear DNA; *mn*, mitochondrial nucleoid DNA; *cn*, chloroplast nucleoid DNA. The scale bars represent 2 *µ*m in the bottom images in **E)**. **F)** A bar graph comparing the percentage of cells with an irregular shape, possessing a single chloroplast and 2 nuclei between the DMSO-treated and the rapamycin-treated cultures at Hour 18. DAPI-stained cells were counted (*n* > 300). The averages and Sd were calculated from biological triplicates.

We then tested whether the rapamycin-inducible protein degradation system is applicable for knocking down endogenous proteins in *C. merolae*. For our initial target, we selected the dynamin-related chloroplast division protein dynamin-related protein 5B (DRP5B) (CMN262C). DRP5B is expressed specifically during S and M phases in *C. merolae* and localizes to the cytosolic side of the chloroplast division site (Miyagishima et al. 2003). A previous study in *C. merolae* showed that the expression of a dominant negative form of DRP5B inhibits chloroplast division (Sumiya et al. 2016). Therefore, it is expected that the knockdown of DRP5B will yield a similar effect. To test this, we generated the transformant referred to as the *DRP5B^RD-SKP1^* line, in which the chromosomal *DRP5B orf* was substituted with *mVenus-4×FLAG-FRB-DRP5B* (*mV-FL-FRB-DRP5B*), and the *HA-FKBP-SKP1* expression cassette was inserted into the intergenic region upstream of the *DRP5B* locus (Fig. 5A).

The immunoblot analyses using anti-DRP5B, anti-FLAG, and anti-HA antibodies confirmed both the substitution of the endogenous DRP5B with the mV-FL-FRB-DRP5B and the heterologous expression of the HA-FKBP-SKP1 in the *DRP5B^RD-SKP1^* strain (Fig. 5B). To synchronize cell cycle progression and the timing of chloroplast division in the culture, the *DRP5B^RD-SKP1^* strain was cultured under the 12-h light and 12-h dark (LD) cycle. The immunoblot analysis showed that mV-FL-FRB-DRP5B is expressed from Hours 9 to 18, peaking at Hour 12 in the culture without rapamycin addition (instead DMSO was added because rapamycin was dissolved in DMSO), as previously shown in the WT (Miyagishima et al. 2003; Yoshida et al. 2010) (Fig. 5C; the onset of the third round of LD was defined as Hour 0). In addition, fluorescent microscopy showed that mV-FL-FRB-DRP5B localized at the chloroplast division site in the *DRP5B^RD-SKP1^* cells from Hours 12 to 15, and then the chloroplast completed the division normally (Fig. 5, C and D). In the *DRP5B^RD-SKP1^* culture, α-tubulin was predominantly expressed from Hours 12 to 18 (Fig. 5C) as previously shown in the WT (Fujiwara et al. 2020), confirming the successful synchronization of the cell cycle under the LD cycle. These results confirmed that the mV-FL-FRB-DRP5B protein functionally complemented DRP5B and did not affect the progression of the chloroplast and cell division cycle, although the level of mV-FL-FRB-DRP5B was somewhat reduced compared with DRP5B (Fig. 5B) probably due to the destabilizing effect of the FRB tag.

Regarding the level of HA-FKBP-SKP1 protein, it decreased during the dark period under the LD cycle (Fig. 5C). The fluctuations in HA-FKBP-SKP1 protein levels are likely attributed to the promoter of *APCC* gene, which was used to express this protein. *APCC* encodes the core linker protein of the phycobilisome, a light-harvesting complex for photosynthesis and, in fact, previous studies have indicated that *APCC* mRNA decreases during the dark period in the LD cycle (Fujiwara et al. 2020).

To knockdown mV-FL-FRB-DRP5B in the *DRP5B^RD-SKP1^* synchronous culture, we added rapamycin at Hour 9 during the LD cycle, just before the peak of the mV-FL-FRB-DRP5B protein level. Immunoblotting using the anti-FLAG antibody confirmed a reduction in the mV-FL-FRB-DRP5B level starting from Hour 12 when compared with the culture without the addition of rapamycin (Fig. 5C). Although there was only 1 dose of rapamycin and FKBP-SKP decreased during the dark period, the mV-FL-FRB-DRP5B level did not recover afterward (Fig. 5C), probably because the synthesis of mV-FL-FRB-DRP5B ceased in accordance with the cell cycle progression.

In the *DRP5B^RD-SKP1^* synchronous culture without rapamycin, cells with a dividing chloroplast and localization of mV-FL-FRB-DRP5B at the division site were observed to be accumulating at Hour 12 (Fig. 5E). Subsequently, most cells completed chloroplast and cell division by Hour 18 (Fig. 5E). In contrast, in the culture treated with rapamycin, chloroplast division did not initiate, and the mVenus fluorescence of mV-FL-FRB-DRP5B was faintly detectable at Hour 12 (Fig. 5E). By Hour 18, abnormally structured cells with 2 separated cytoplasmic regions and a single undivided chloroplast had accumulated (Fig. 5E). The DAPI staining and immunofluorescence with anti-mitochondrial porin antibody showed that these abnormal *DRP5B^RD-SKP1^* cells at Hour 18 contained 2 sets of the nucleus and mitochondrion but only 1 chloroplast, in contrast to normal telophase cells that contain 2 sets of the nucleus, mitochondrion, and chloroplast (Fig. 5E). At Hour 18, the abnormal cells accounted for ∼76% of the population in the *DRP5B^RD-SKP1^* synchronous culture with rapamycin while they were rarely observed in the culture without rapamycin (Fig. 5F). These results are consistent with the previous study that expressed a dominant negative form of DRP5B in *C. merolae* (Sumiya et al. 2014, 2016) and indicate that mitochondrial division and chromosome segregation progressed even when chloroplast division was blocked at the phase where DRP5B was recruited to the division site.

### Rapamycin-induced knockdown of the endogenous transcription factor E2F

Finally, we investigated whether the developed inducible knockdown method could also be applied to nuclear proteins. We chose E2F (CMT067C) to be knocked down, which is known to control the G1/S transition as described above (Fig. 2A; De Veylder et al. 2002; Miyagishima et al. 2014; Chen et al. 2009) and whose nuclear localization has been confirmed in *C. merolae* (Miyagishima et al. 2014). E2F activates the transcription of S-phase genes such as those for chromosomal replication and cell cycle regulation (Chen et al. 2009). Additionally, it has been reported that E2F is also involved in various other cellular processes such as differentiation, metabolism, and mitochondrial functions in mouse (*Mus musculus*), *Drosophila* (*D. melanogaster*), and *Arabidopsis* (*Arabidopsis thaliana*) (Blanchet et al 2011; Ambrus et al 2013; Yao et al. 2018; Mäkelä and Toppar 2022).

**Figure 6.**
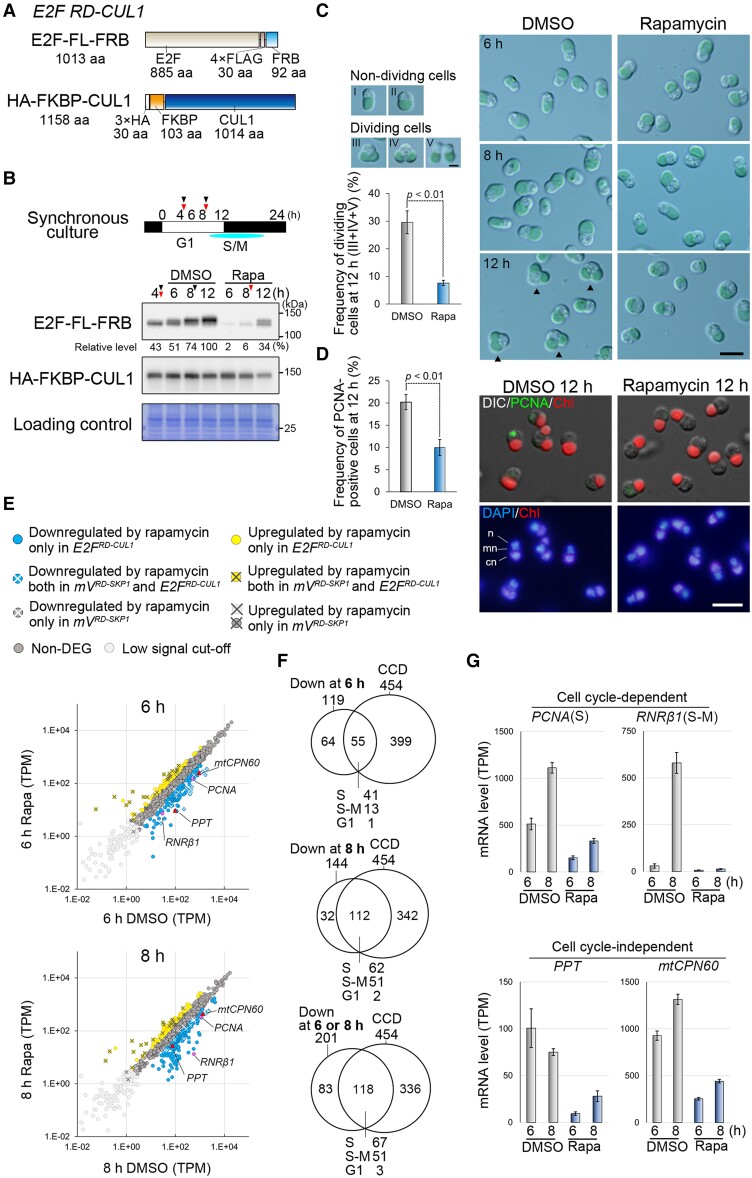
Investigation of the E2F-dependent transcriptome using rapamycin-induced degradation of E2F. **A)** The structures of E2F-FL-FRB and HA-FKBP-CUL1 expressed in the *E2F^RD-CUL1^* knock-in strain. 4×FLAG (FL) and FRB were fused to the carboxy-terminus of E2F for immunological protein detection and rapamycin-inducible interaction with HA-FKBP-CUL1, respectively. The 6×G peptide linker was inserted between FL and FRB. **B)** Immunoblotting with the anti-FLAG antibody confirmed rapamycin-induced degradation of E2F-FL-FRB protein in the *E2F^RD-CUL1^* strain. DMSO as a negative control, or 500 nm rapamycin was added at Hour 4 under LD. The white and black bar indicates light and dark periods, respectively. “G1” and “S/M” below the bar indicate the G1 phase and the S and M phases of the cell cycle, respectively. The light blue line below the bar represents the period from the S to M phases. The immunoblotting of HA-FKBP-CUL1 with an anti-HA antibody is also shown. The CBB-stained PVDF membrane is also shown as a loading control. The predicted molecular weight based on the amino acid sequence of E2F-FL-FRB and HA-FKBP-CUL1 is 111 and 127 kDa, respectively. **C)** Comparison of the cell cycle progression between the DMSO and rapamycin-treated *E2F^RD-CUL1^* synchronous cultures. The cell cycle stage was divided into I (vertically elongated cells; early G1), II (enlarged cells; late G1), III (cells with a dividing chloroplast; M, prophase), IV (cells with 2 divided chloroplasts; M, metaphase), and V (cells during cytokinesis) according to Fujiwara, Tanaka, et al. (2013), and the percentage of the sum of cells in Stages III, IV, and V is compared at Hour 12. The values represent the mean of 3 independent cultures, and the bars indicate the Sd (*n* > 200). *P* < 0.01 (Student’s *t*-test). The scale bar represents 2 *µ*m in the 5 typical cell images showing the cell cycle phases, which are above the bar graph, and 5 *µ*m in the 6 images that are to the right of the bar graph. The arrowheads indicate dividing cells. **D)** Immunofluorescence microscopy comparing the frequency of the cells expressing PCNA (S phase) at Hour 12 between the DMSO and rapamycin-treated *E2F^RD-CUL1^* synchronous cultures. PCNA protein was detected with the anti-PCNA antibody and DNA was stained with DAPI. The values represent the mean of 3 independent cultures, and the bars indicate the Sd (*n* > 200). *P* < 0.01 (Student's *t*-test). *n*, nuclear DNA; *mn*, mitochondrial nucleoid DNA; *cn*, chloroplast nucleoid DNA. The scale bar represents 5 *µ*m in all images in **D)**. **E)** Scatter plots comparing the transcriptome (RNA-seq results) between the DMSO and rapamycin-treated *E2F^RD-CUL1^* cultures. Transcripts per kilobase million (TPM) values were averaged from 4 biological replicates. Genes downregulated or upregulated (FDR of <0.01 and a log2 fold-change of >+1 or <−1) in the presence of rapamycin are represented by blue or yellow circles, respectively. Above the graphs, there is an explanation of what each symbol represents. In brief, the blue and yellow circles without crosses indicate genes that were downregulated and upregulated by the degradation of E2F. **F)** Venn diagrams classifying the genes downregulated by the rapamycin-induced degradation of E2F as either CCD or cell cycle–independent ones. Four hundred forty-five CCD genes in *C. merolae* were identified in a previous study (Fujiwara et al. 2020). **G)** Bar graphs showing mRNA levels of *PCNA*, *RNRβ1*, phosphate/*PPT*, and mitochondrial 60-kDa chaperonin (*mtCPN60*), which were downregulated by the rapamycin-induced degradation of E2F. Of these, *PCNA* and *RNRβ1* are CCD, while *PPT* and *mtCPN60* are not. These genes were also indicated in C by the red or pink symbols. The values represent the mean of 4 independent RNA sequencing, and the bars indicate the Sd.

To conditionally knock down E2F and examine its impact on the transcriptome, we generated a transformant referred to as the *E2F^RD-CUL1^* strain. In this strain, the chromosomal E2F *orf* was substituted with *E2F-4×FLAG-FRB* (*E2F-FL-FRB*), and the *HA-FKBP-CUL1* gene cassette was inserted upstream of the *URA* gene locus using a previously developed cotransformation method (Fujiwara et al. 2021) (Fig. 6A). The immunoblot analysis confirmed the expression of E2F-FL-FRB and HA-FKBP-CUL1 proteins in the *E2F^RD-CUL1^* strain (Fig. 6B).

The *E2F^RD-CUL1^* culture was synchronized under the LD cycle. At Hours 4 and 8, rapamycin or DMSO (used as a negative control) was added to the culture. The second addition was applied to sustain the activity of rapamycin as shown in Supplementary Fig. S2. The cells were collected at Hours 6 to 12 (Fig. 6B). These time points were selected because the mRNA levels of S-phase genes begin to rise around Hour 8 and the frequencies of S- and M-phase cells peak around Hour 12 in the WT *C. merolae* under the LD cycle (Fujiwara et al. 2020). The immunoblot analysis showed that the addition of rapamycin successfully reduced E2F-FL-FRB levels at Hours 6 to 12 compared with the culture without rapamycin (Fig. 6B). Thus, the results show that the rapamycin-inducible protein degradation system is applicable also to nuclear proteins.

To evaluate the influence of the reduction of E2F level on the cell cycle progression, we examined the frequencies of dividing cells (M-phase; Fig. 6C) based on the shape of the cell and chloroplast according to Fujiwara, Tanaka, et al. (2013). In addition, we quantified cells in the S phase by immunofluorescence microscopy using the anti-proliferating cell nuclear antigen (PCNA) antibody as an S-phase marker (Fujiwara, Tanaka, et al. 2013; Fig. 6D) at Hour 12 in the LD cycle. When rapamycin was added, the frequencies of both M-phase cells in the culture at Hour 12 (7%) significantly decreased compared with those in the culture with DMSO (29%). PCNA-positive S-phase cells also decreased in the culture with rapamycin (9%) compared with that with DMSO (20%) (Fig. 6D). Thus, the results demonstrate that rapamycin-induced reduction of E2F level resulted in delay of G1/S transition.

The transcriptome analysis by RNA-seq showed that 119 and 144 (201 in total) genes were downregulated at Hours 6 and 8, respectively (Fig. 6E; Supplementary Table S1; FDR < 0.01, log2 fold-change < −1). Three genes that were upregulated at Hour 6 but downregulated at Hour 8 have been excluded. Additionally, genes that were exclusively downregulated due to the side effect of FKBP and rapamycin (Supplementary Fig. S3), regardless of E2F degradation, have also been excluded. As expected, these downregulated genes included typical S-phase genes such as *PCNA* and *RNRβ1* (ribonucleotide-diphosphate reductase small subunit) (Fujiwara et al. 2020), consistent with the role of E2F as a G1/S regulator (Fig. 2A).

Then, we compared the results of the E2F knockdown with the list of genes exhibiting cell cycle–dependent (CCD) expression that were identified in a previous study (454 CCD genes; Fujiwara et al. 2020). The 201 genes downregulated by E2F knockdown (at either Hour 6 or 8) were categorized into 118 CCD genes and 83 non-CCD genes (Fig. 6F). The 118 CCD genes included 67 S-phase genes and 48 S–M-phase genes, as classified in the previous study (Fujiwara et al. 2020). Additionally, among these CCD genes, 3 were identified as G1-phase genes (Fujiwara et al. 2020). Specifically, they were CTS synthase, which converts UTP to CTP, and 2 genes with unknown functions (Supplementary Table S1). The 83 non-CCD genes included 4 genes encoding transporters, such as phosphate/phosphoenolpyruvate translocator (PPT), 8 genes encoding molecular chaperones, such as mitochondrial chaperonin HSP60 (mtCPN60), and genes related to metabolism (Fig. 6G; Supplementary Table S1). At this point, the biological significance of the expression of these genes affected by E2F, as well as whether their expression is directly or indirectly influenced by E2F, remains unclear. Further studies would uncover the relationships between the cell cycle and metabolism.

The transcriptome analysis also identified 76 and 92 (120 in total) genes were upregulated at Hours 6 and 8, respectively by the E2F knockdown (Fig. 6E; Supplementary Table S1; FDR < 0.01, log2 fold-change > 1). The most highly upregulated gene was CMG022C of unknown function, which is unique to *C. merolae*. In the remaining genes, the degree of expression variation was not notably high, and no particular functional features were identified (Fig. 6E; Supplementary Table S1).

## Discussion

In this study, we have successfully developed a constitutive and inducible protein-knockdown system in the unicellular red alga *C. merolae*, where RNAi is not applicable. Until now, the inactivation of essential proteins in *C. merolae* has been achieved either by expressing a dominant negative form of a protein using endogenous heat shock promoter (DRP5B in Sumiya et al. 2016) or by repressing the transcription of a gene coupled with a promoter related to nitrate assimilation, which is turned off in the presence of ammonium in the medium (CDKA in Fujiwara et al. 2020). However, the former method is only applicable to a protein for which information about a dominant negative mutation is available. As for the latter method, it is only feasible for constitutively expressed genes like *CDKA*, because it requires replacing the promoter of the target gene on the chromosome with the promoter of nitrate assimilation genes, which constitutively express the target gene in the nitrate medium without ammonium (Fujiwara et al. 2020). Additionally, both heat shock and the shift from nitrate to ammonium as the nitrogen source have substantial side effects because these treatments can substantially alter cellular physiology (Imamura et al. 2009; Kobayashi et al. 2014). Compared with these previous methods, the rapamycin-inducible protein-knockdown system is, in principle, applicable to any cytosolic and nuclear proteins and, as demonstrated here, has minimal side effects. Therefore, this system will facilitate studies on essential genes and mechanisms in *C. merolae*.

While the protein-knockdown system offers several advantages, the system developed here still possesses certain limitations. These include the following: (i) Because of the short duration of rapamycin action in *C. merolae* culture, repetitive doses are necessary to maintain a reduced level of the target protein over an extended period (Supplementary Fig. S2). (ii) Although the transcriptome analysis suggests that the side effects are not substantially pronounced (Supplementary Fig. S3), caution may be needed when analyzing metabolic pathways. (iii) The level of the target protein is reduced before rapamycin treatment due to the destabilizing effect of the FRB tag when compared with the WT (Fig. 5B).

To overcome limitation (i), a chemical screening of a rapamycin derivative that sustains the effect over a long period in *C. merolae* culture is required. To address limitation (ii), rapamycin analogs, such as C20-methallylrapamycin (C20-MaRap; Stankunas et al. 2003), would be effective. C20-MaRap exhibits substantially lower affinity for the WT FRB domain of TOR but a higher affinity for FRB mutants K2095P-T2098L-W2101F and K2095P-W2101F. Therefore, this analog does not affect endogenous TOR (Stankunas et al. 2003). Unfortunately, the potential use of C20-MaRap could not be assessed in this study for *C. merolae* because it was not available in our research environment at this stage. It should be noted that the rapamycin-based system can be applied to *C. merolae* because the endogenous FKBP is insensitive to rapamycin, in contrast to FKBP in other organisms. As a result, rapamycin does not affect TOR in the WT *C. merolae* (Imamura et al. 2013).

At this point, no inducible protein-knockdown system utilizing a dimerization-inducing chemical, such as rapamycin utilized in this study, is available in other photosynthetic organisms. In cyanobacteria, a protein-knockdown system has been developed in which a target protein is fused to a protein degradation tag and degraded by conditionally expressed LON protease (Sakkos et al 2021). In *Arabidopsis* and maize, a method has been developed to inactivate target proteins by inducing their aggregation through the estradiol-induced expression of an intrinsic aggregation-prone region (APR) within the target protein exogenously (Betti et al. 2016, 2018). However, this method is only applicable to proteins that contain an APR. Besides these methods, during the course of revising this paper, the E3-DART protein-knockdown system was reported in *Nicotiana benthamiana* and *Arabidopsis*. This system utilizes the interaction between the Salmonella-secreted protein H1 (SspH1) and the HR1b domain of protein kinase N1 (PKN1), a target of SspH1 in humans. The SspH1 protein possesses E3 ubiquitin ligase activity and binds to a target protein fused with HR1b, resulting in the degradation of the target protein. SspH1 can be transcriptionally induced using dexamethasone to conditionally knock down a target protein (Huang and Rojas-Pierce 2024). However, the systems that utilize the LON protease, APRs, or SspH1 rely on the transcription and translation of these proteins, thus requiring a longer duration to knock down a target protein after induction compared with the addition of dimerization-inducing chemicals.

Model photosynthetic eukaryotes, such as the land plant *A. thaliana* and the green alga *C. reinhardtii*, are sensitive to rapamycin (Crespo et al. 2005; Xiong and Sheen 2012). However, the adoption of a rapamycin analog, which does not heterodimerize the endogenous FKBP and FRB domain of TOR kinase in algae and plants, would allow the application of the system developed in this study to other model organisms, including *C. reinhardtii* and *A. thaliana*. In this regard, the rapamycin analogs such as C20-MaRap (Stankunas et al. 2003) or AP21967 (Bayle et al. 2006; as described earlier, heterodimerizing FRB^T2098L^ but not the WT FRB with FKBP) would be applicable. To address limitation (iii), the use of the Stabilon tag (Rethi-Nagy et al. 2022) counteracts the destabilizing activity of FRB to a certain extent (Supplementary Fig. S5). Another potential solution is to exchange FRB and FKBP. If FKBP is fused to the target protein and FRB is fused to SKP1 or CUL1, the issue might be resolved.

Thus far, several other inducible systems for protein knockdown using dimerization-inducing chemicals have been developed in yeast and mammalian cell lines. Notable examples include the auxin-inducible degron (AID) system (Nishimura et al. 2009; Yesbolatova et al. 2020) and the HaloPROTAC system (Tovell et al. 2019). In the AID system, the AID domain, derived from the *A. thaliana* auxin-responsive protein IAA17, is fused to the target protein, and the F-box protein TIR1, derived from *Oryza sativa*, is introduced to the SCF E3 ligase. In the transformant, natural and synthetic auxins induce the dimerization of the AID-fused target protein and the TIR1-bound SCF E3 ligase, leading to the degradation of the target protein through the ubiquitin-proteasome pathway (Nishimura et al. 2009; Yesbolatova et al. 2020). However, we could not apply the AID system to *C. merolae* due to the toxicity of auxins to the cells, although the cause of this toxicity is currently unknown. Alternatively, in the HaloPROTAC system, the Halo tag is fused to the target protein, and a chimeric chemical composed of the Halo-tag ligand and VHL ligand is used to bind the target protein to CRL2–VHL E3 ligase (Cul2 E3 ligase complexed with RBX1, EloBC, and VHL) (Tovell et al. 2019). Other inducible protein degron systems (e.g. Bromo tag and dTAG; Nabet et al. 2018; Bond et al. 2021; Kanemaki 2022) operate on a similar principle; however, they differ in terms of the combinations of dimerization inducers, tags for target proteins, and E3 ligases utilized. To assess the suitability of the HaloPROTAC or other systems to *C. merolae* or other photosynthetic eukaryotes, it is necessary to examine the suitability of the E3 ligase utilized and the permeability of the dimerization inducer.

## Materials and methods

### Culture conditions of *C. merolae*

*C. merolae* 10D WT (NIES-3377), its transformants, and the uracil-auxotrophic mutant M4 (a derivative of *C. merolae* 10D, which has a mutation in the *URA* gene; Minoda et al. 2004) were cultured in MA2 medium (an inorganic medium; Ohnuma et al. 2008). For the M4 strain, the medium was supplemented with 0.5-mg/mL uracil. All strains were maintained in 20 mL of the medium in 25-cm^2^ tissue culture flasks (90026; TPP Techno Plastic Products AG) in the light (30 *µ*mol m^−2^ s^−1^) at 42 °C in an incubator shaker (BR-43FL; TITEC, Japan) with agitation at 130 rpm.

For immunoblotting, microscopy, measuring cell volume and transcriptome analysis, all strains examined were initially diluted to give an OD750 of 0.2 in 50 mL of MA2 medium in 100-mL test tubes (IWAKI, Japan) then cultured with aeration (400 mL ambient air min^−1^) at 42 °C under continuous light (70 *μ*mol m^−2^ s^−1^) or the LD cycle for 2 or 3 d.

For rapamycin treatment, 1 and 10 mm stock solutions of rapamycin (FUJIFILM Wako Pure Chemical) in DMSO were prepared. Final concentrations of 10, 50, and 100 nm in cultures were achieved by adding 1 mm rapamycin solution with dilutions of 1:100,000, 1:20,000, and 1:10,000, respectively. For higher concentrations (500, 1,000, and 2,500 nm), the 10 mm rapamycin solution was used with dilutions of 1:20,000, 1:10,000, and 1:4,000, respectively. To draw growth curves of WT and *mV^RD-SKP1^* cultures in the absence (DMSO only) or presence of rapamycin, the OD750 of 3 cultures each of WT and the same clone of *mV^RD-SKP1^* were monitored for 14 d.

### Preparation of *C. merolae* transformants

To generate *C. merolae* transformants, we first constructed plasmid containing the construct for homologous recombination-mediated genetic modification. Then, the construct was amplified by PCR, and the resultant linear DNA was introduced into *C. merolae* 10D or M4 by a PEG-mediated method (Ohnuma et al. 2008). The DNA sequences for the constructs were either amplified from *C. merolae* genomic DNA by PCR or artificially synthesized and assembled using the In-Fusion Snap Assembly Master Mix (Takara Bio, Japan). The plasmid sequences are shown in Supplementary Table S2.

The DNA constructs for *C. merolae* transformation were amplified from the corresponding plasmids by PCR with the primer set (forward 5′-cgttgtaaaacgacggccagt-3′ and reverse 5′-acaatttcacacaggaaacagctatgac-3′) and then purified using a NucleoSpin Gel and PCR Clean-up (Takara). A total of 2 to 4 *µ*g of the purified DNA was used for transformation. The DNA introduction and selection of transformants were performed as previously described (Fujiwara et al. 2015, 2017, 2021).

The DNA constructs containing the gene cassettes of *mVenus*, *mVenus-FRB*, *mVenus-3×FRB*, mVenus-FRB*, mVenus-3×FRB*, *mVenus-FRB/HA-FKBP-SKP1*, *mVenus-FRB/HA-FKBP-CUL1*, *mVenus-FRB/HA-FKBP-RBX1*, and *mVenus-FRB^T75L^/HA-FKBP-SKP1* with the *URA* selectable marker were inserted into the upstream of *URA* locus of the *C. merolae* M4 strain to generate the *mVenus*, *mVenus-FRB*, *mVenus-3×FRB*, mVenus-FRB*, mVenus-3×FRB*, *mV^RD-SKP1^*, *mV^RD-CUL1^*, *mV^RD-RBX1^*, and *mV^RDTL-SKP1^* strains, respectively. To generate the *RB* variant and *RB-KO* strains, DNA constructs with sequences of *HA-RB*, *HA-FRB-RB*, *HA-3×FRB-RB*, HA-FRB*-RB, *HA*-3×FRB*-RB, and *RB-KO*, along with the *CAT* selectable marker, were integrated to the *RB* locus of *C. merolae* 10D WT. To generate the *DRP5B^RD-SKP1^* strain, the DNA construct containing the gene cassettes of *DRP5B/HA-FKBP-SKP1* with the *URA* marker was integrated to the *DRP5B* locus of *C. merolae* M4. To generate the *E2F^RD-CUL1^* strain, we simultaneously introduced the DNA construct containing the gene cassettes of *HA-FKBP-CUL1* with the *URA* marker and the construct containing the *E2F-FL-FRB* with the *CAT* marker into *C. merolae* M4 according to the cotransformation procedure previously described (Fujiwara et al. 2021). Subsequently, the transformants were selected in uracil-free medium supplemented with chloramphenicol.

### Immunoblotting

The *C. merolae* cells were harvested by centrifugation at 2,000 × *g* for 5 min at 4 °C. The cell pellets were lysed with the sample buffer (2% [*w*/*v*] SDS, 62 mm Tris-HCl, pH 6.8, 100 mm DTT, 10% [*w*/*v*] glycerol, and 0.01% [*w*/*v*] bromophenol blue) and then incubated at 95 °C for 5 min. After centrifugation at 20,000 × *g* for 5 min, the protein concentration in the supernatant was measured using an XL-Bradford kit (Aproscience). The total proteins (6 *µ*g) were separated on polyacrylamide gels and then transferred to PVDF membranes (immobilon, Millipore). The membranes were blocked with 5% *w*/*v* dry skim milk dissolved in TTBS (20 mm Tris-HCl pH7.5, 200 mm NaCl, and 0.1% *v*/*v* Tween 20). The primary antibodies were diluted in Bullet ImmunoReaction Buffer (Nacalai Tesque) and used at the following dilutions: anti-GFP (for detection of mVenus; dilution of 1:2,000; clone JL-8, Takara), anti-eEF-2 (1:4,000; Nishida et al. 2005), anti-HA (1:5,000; clone 16B12, BioLegend), anti-FLAG (1:4,000; clone M2, Sigma), anti-α-tubulin (1:2,000; clone B-5-1-2, Sigma), anti-RB (1:4,000; Miyagishima et al. 2014), and anti-DRP5B (1:3,000; Miyagishima et al. 2003). As secondary antibodies, HRP-conjugated anti-mouse or anti-rabbit IgG (1:40,000; Thermo Fisher Scientific) was used. The signals were detected using SuperSignal West Atto Ultimate Sensitivity Substrate (Thermo Fisher Scientific) and ChemiDoc Touch Imaging System (BIO-RAD). The signal intensities of target proteins were quantified using ImageJ (https://imagej.nih.gov/ij/download.html).

Technical triplicates of immunoblotting were performed to determine the relative signal intensity and Sd of mVenus and different FRB-fused mVenus proteins (Fig. 1). Additionally, immunoblotting using 2 independent transgenic clones of each strain was conducted to assess the relative signal intensity of RB and various versions of modified RB proteins (Fig. 2). Changes in the relative signal intensity of mVenus-FRB were analyzed using 3 independent clones of *mV-FRB*, *mV^RD-SKP1^*, *mV^RD-CUL1^*, and *mV^RD-RBX1^* (Fig. 4B). Furthermore, alterations shown in Fig. 4, D and E were assessed through immunoblotting using samples from biological triplicates subjected to rapamycin treatment of the same clone. The relative signal intensity of mV-FL-FRB-DRP5B (Fig. 5, B and C), E2F-FL-FRB, and PCNA (Fig. 6B) proteins, along with their changes in the LD, was evaluated in a single experiment. Additionally, the relative signal intensity of the mVenus-FRB (Supplementary Fig. S2) and the mVenus-FRB^T75L^ (Supplementary Fig. S4) proteins was also evaluated in a single experiment. To evaluate changes in Supplementary Fig. S5, the same clones were analyzed 3 times for both the *mVenus* and *mV^RD-CUL1^* strains, whereas 3 independent transformed clones were analyzed for the *Stab-mV^RD-CUL1^* and *mV-Stab^RD-CUL1^* strains (Supplementary Fig. S5).

### Microscopy

The *C. merolae* cells were observed using differential interference optics with a microscope (IX71, Olympus) or (BX51, Olympus).

The mVenus fluorescence and autofluorescence of the chloroplasts were captured using a fluorescence microscope (IX71, Olympus) equipped with an sCMOS camera system (Zyla 4.2 PLUS, Andor), a mercury lamp (USH-1030L, Olympus), and a 60× oil objective lens (UPlanApo N, Olympus). The filter sets LF514-C-U03 (Semrock) and U-MWIG3 (Olympus) were used for mVenus fluorescence and chloroplast autofluorescence, respectively. Exposure times were set to 1 s for mVenus and 0.01 s for chloroplast autofluorescence, respectively.

Immunostaining of the mitochondrion was conducted using an anti-mitochondrial porin antiserum raised in guinea pigs as the primary antibody at a 1:500 dilution as previously described (Fujiwara et al. 2010). PCNA immunostaining was conducted using an affinity purified anti-PCNA antibody raised in rabbits as the primary antibody, at a final concentration of 1 *µ*g/mL as previously described (Fujiwara, Tanaka, et al. 2013). As the secondary antibody, anti-guinea pig IgG conjugated with Alexa Fluor 555 (Thermo Fisher Scientific) and anti-rabbit IgG conjugated with Alexa Fluor 488 (Thermo Fisher Scientific) were used. Additionally, DNA was counterstained with 1-*µ*g/mL DAPI. To detect DAPI, chloroplast, Alexa Fluor 555 and Alexa Fluor 488 fluorescence, band-pass filter sets U-MWU2, U-MWIG3 (Olympus), XF37 (Omega), and U-MWIBA3 (Olympus) were used, respectively. The images were captured using a fluorescence microscope (BX51, Olympus) equipped with a digital CCD camera system (DP71, Olympus), a mercury lamp (USH-1030L, Olympus), and a 60× oil objective lens (UPlanApo N, Olympus). Exposure times were set to 0.1 s for DAPI-stained DNA, 0.5 s for Alexa Fluor 555-labeled mitochondrial porin, 1 s for mVenus-FL-FRB-DRP5B and Alexa Fluor 488-labeled PCNA, and 0.01 s for chloroplast autofluorescence. The proportions of S/M phase cells and PCNA-positive cells were calculated based on 3 biological replicates. Statistical analysis was performed using Student's *t*-test.

### Cell size measurement

The cell size of *C. merola*e 10D (WT) and 2 clones of each *RB*-modified strain was measured using a Multisizer 4e Coulter counter (Beckman Coulter). Statistical analysis was performed using Student's *t*-test.

### Transcriptome analysis

Cells were harvested by centrifugation at 2,000 × *g* for 5 min at 4 °C., frozen in liquid nitrogen, and stored at −80 °C until use. Total RNA was extracted following the Trizol/RNeasy hybrid protocol (Trizol, Life Technologies; RNeasy Mini Kit, Qiagen) according to the manufacturer's instructions. To construct a cDNA library of 150 bp, 100 ng of total RNA were used. Paired-end sequencing was performed using the Illumina sequencing platform (NovaSeq 6000) according to the manufacturer's instructions (Illumina). The coding sequence was used as the reference for the *C. merolae* transcripts. To obtain gene expression scores, one side of the trimmed paired-end reads was mapped to the reference by Bowtie2 ver. 2.3.4.1 (Langmead and Salzberg 2012). SAMtools ver. 1.8 (Li et al 2009), BEDtools ver. 2.19.1 (Quinlan and Hall 2010), and R ver. 3.5.3 (Ihaka and Gentleman 1996) were used to calculate the number of reads mapped to the contigs (raw count).

To compare changes in the transcriptome, the count data were analyzed by TCC ver. 1.40.0 (Sun et al. 2013) in R using biological quadruplicated experiments. Genes were identified as DEGs only when FDR was <0.01 and the absolute value of logFC was >1.

### Accession numbers

The RNA sequence data from this article can be found in the GenBank/EMBL data libraries under accession numbers: BioProject accession PRJDB16885; BioSample accession nos. SAMD00653757 to SAMD00653788; and DRA accession no. DRA017302.

## Supplementary Material

kiae316_Supplementary_Data

## Data Availability

The RNA-seq data obtained in this study have been deposited in the DNA Data Bank of Japan (DDBJ)/EMBL/GenBank (BioProject accession PRJDB16885; BioSample accession nos. SAMD00653757 to SAMD00653788; and DRA accession no. DRA017302). All other data are included in the manuscript and/or Supplementary Table S1. The nucleotide sequences of the linear DNA introduced into *C. merolae* were shown in Supplementary Table S2.

## Supplementary data

The following materials are available in the online version of this article.

**Supplementary Figure S1**. Comparison of the distribution of cell size in the WT, *HA-RB*, and the 4 different types of *HA-FRB-RB* cultures.

**Supplementary Figure S2**. Evaluation of the duration of rapamycin-induced degradation of the mVenus-FRB protein and the effect of additional rapamycin doses.

**Supplementary Figure S3**. Evaluation of the effect of rapamycin on cell growth and transcriptome in the WT and *mV^RD-SKP1^*.

**Supplementary Figure S4**. The rapamycin analog AP21967 was ineffective in the rapamycin-inducible protein degradation system in *C. merolae*.

**Supplementary Figure S5.** The addition of a Stabilon tag improved the stability of mVenus-FRB.

**Supplementary Table S1**. List of *C. merolae* genes, edgeR output values, and TPM values in the WT and *mV^RD-SKP1^* culture 2 h after the addition of rapamycin or DMSO, and in the *E2F^RD-CUL1^* culture at Hours 6 and 8 under the LD cycle with or with or rapamycin.

**Supplementary Table S2**. List of nucleotide sequences of the linear DNA introduced into *C. merolae* in this study.
