# Supplementary material for: Development of a rapamycin-inducible protein-knockdown system in the unicellular red alga Cyanidioschyzon merolae
Source: Plant Physiol. 2024 Jun 4;196(1):77–94. doi: 10.1093/plphys/kiae316 (PMC11376382; doi:10.1093/plphys/kiae316)
Supplement: kiae316_Supplementary_Data [file kiae316_supplementary_data.zip › Supplementary Figures S1-5.pdf]

Supplementary Figure S1

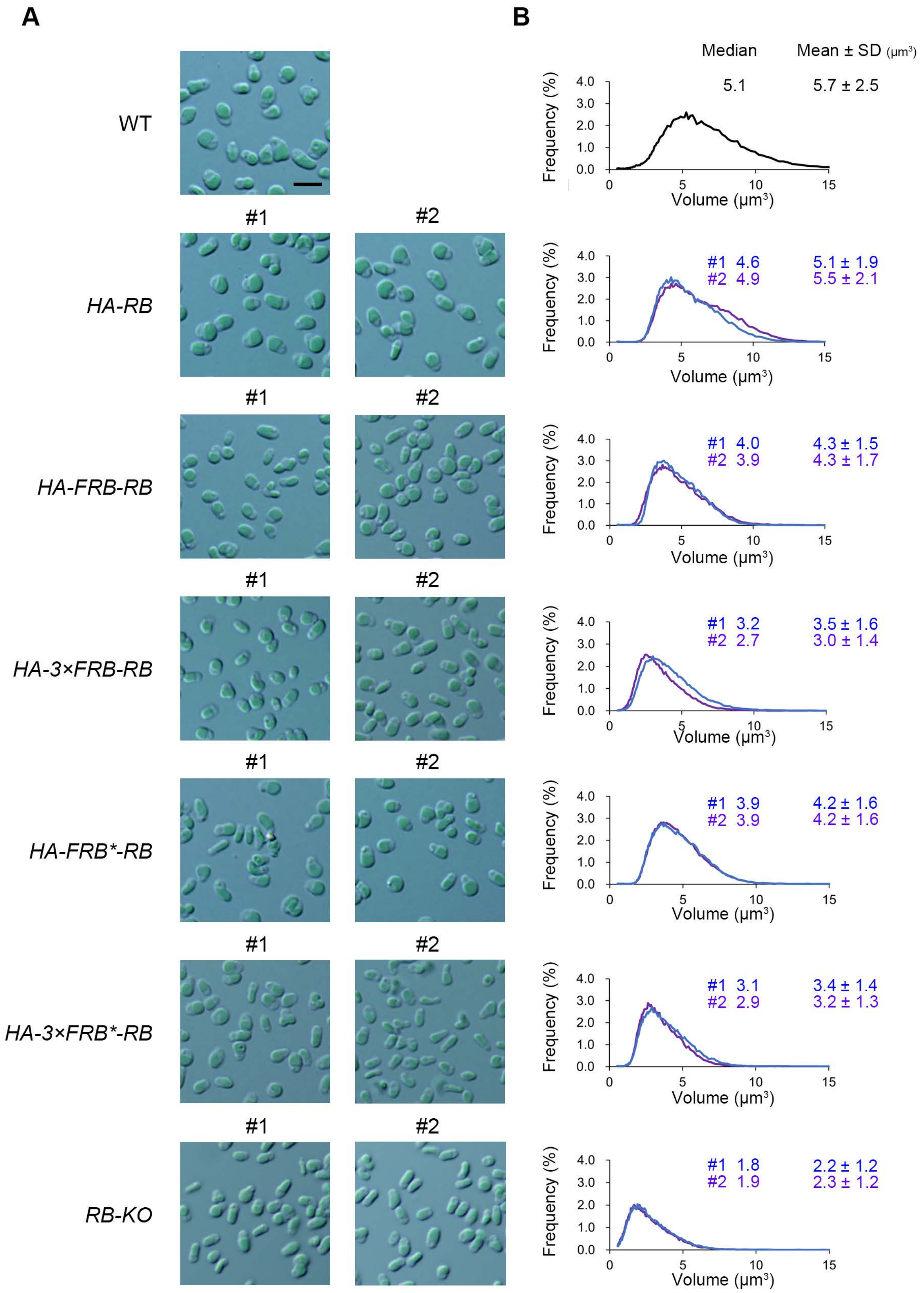

Supplementary Figure S1. Comparison of the distribution of cell size in the wild-type, *HA-RB*, the four different types of *HA-FRB-RB*, and *RB-KO* cultures.

(A) DIC images of the cells in respective cultures. The cells were cultured asynchronously in the light and log-phase cultures were analyzed. #1 and #2 were biological replicates utilizing independently obtained two clones. Bar = 5 μm in all images in A.

(B) Histograms showing the distribution of the cell size in the respective cultures.

Supplementary Figure S2

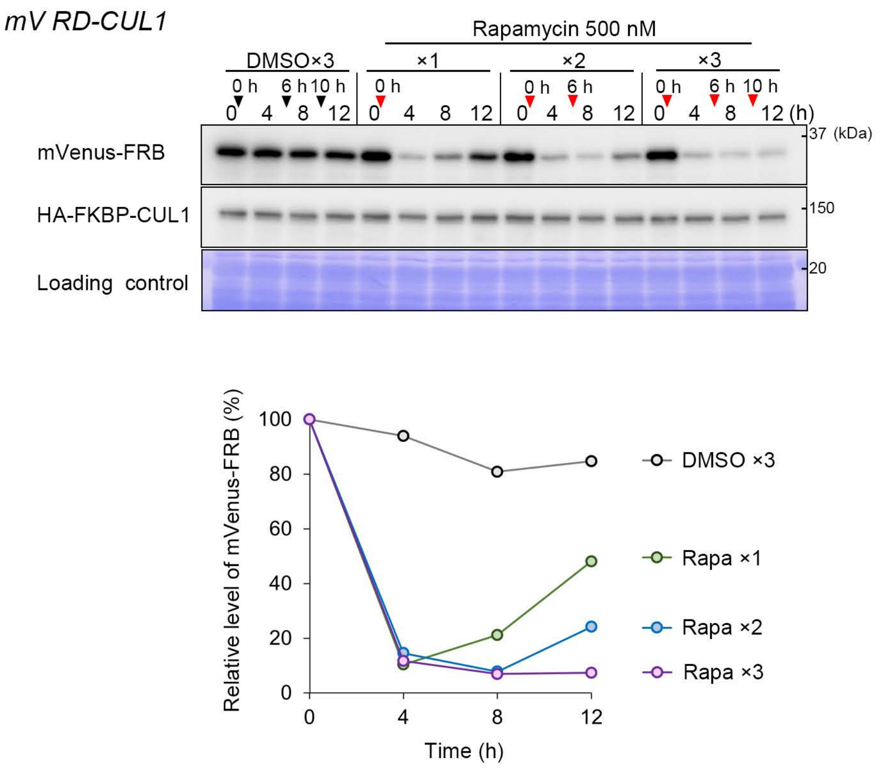

Supplementary Figure 2. Evaluation of the duration of rapamycin-induced degradation of the mVenus-FRB protein and the effect of additional rapamycin doses.

Immunoblotting with the anti-GFP antibody showed that the mVenus-FRB protein level in the *mV*$^{RD\text{-}CUL1}$ strain decreased by the addition of rapamycin (500 nM) for 4 h, but after that gradually recovered (1×). An additional rapamycin dose (500 nM) 6 h after the first addition suppressed the recovery of the protein level until 8 h, but not 12 h after the first addition (2×). The third dose (500 nM) 10 h after the first addition once again impeded the recovery 12 h after the first addition (3×). Also shown is an immunoblotting with the anti-HA antibody showing constant expression of the HA-FKBP-CUL1 protein in the *mV*$^{RD\text{-}CUL1}$ culture. The CBB-stained PVDF membrane is shown as a loading control. The graph shows the change in the mVenus-FRB protein level. The signal intensities of the mVenus-FRB bands on the immunoblot were quantified with ImageJ software, with the hour 0 (just before the first dose of rapamycin or DMSO) set to 100%.

Supplementary Figure S3

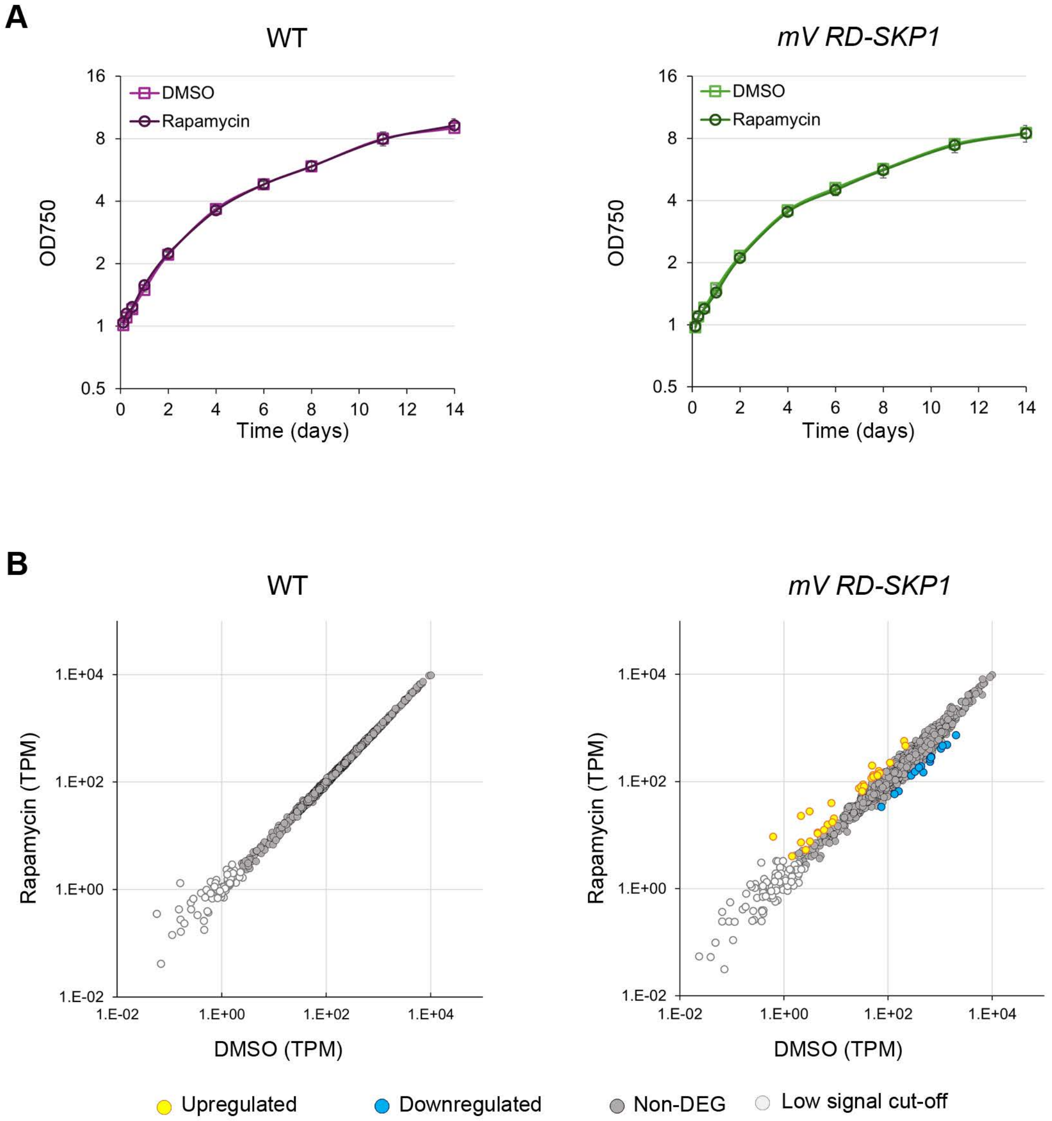

Supplementary Figure S3. Evaluation of the effect of rapamycin on cell growth and transcriptome in the wild-type and *mV$^{RD\text{-}SKP1}$* strains.

(A) Growth curves of WT and *mV$^{RD\text{-}SKP1}$* cultures in the absence (DMSO only) or presence of rapamycin. After the addition of 500 nM rapamycin or DMSO, the OD750 of the cultures was monitored for 14 days. The values represent the mean of three biological replicates, and the bars indicate the standard deviation.

(B) Comparative RNA-seq analyses of WT and *mV$^{RD\text{-}SKP1}$* cultures in the absence or presence of rapamycin. The cells were harvested for the RNA-seq analyses 2 h after the addition of 500 nM rapamycin or DMSO. TPM (Transcripts Per Kilobase Million) values were averaged from four biological replicates.The yellow and blue circles indicate genes that are up- and downregulated (FDR < 0.01, a log2 fold-change >1 or <-1) by the rapamycin treatment.

Supplementary Figure S4

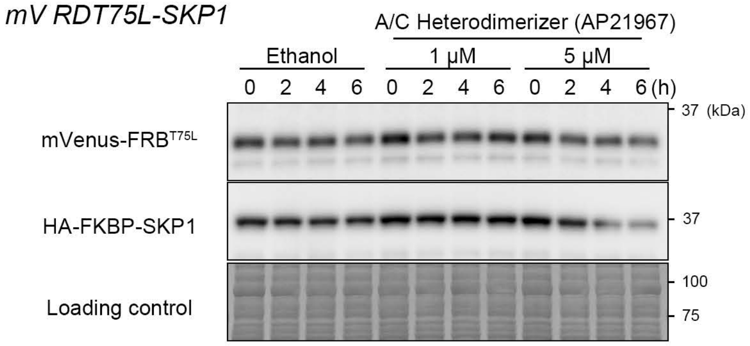

Supplementary Figure S4. The rapamycin analog AP21967 was ineffective in the rapamycin-inducible protein degradation system in *C. merolae*. AP21967 (A/C Heterodimerizer) facilitates heterodimerization between the FRB variant FRB^{T75L} (corresponding a mammalian TOR1T2098L mutation) and FKBP, resulting in a significant reduction in cytotoxicity compared to rapamycin. AP21967 was added to the $mV^{RDTL\text{-}SKP1}$ culture at final concentrations of 1 or 5 µM for 2, 4, and 6 h. Ethanol, the vehicle for AP21967, was added to the control culture. Immunoblotting with the anti-GFP antibody showed that the mVenus-FRB protein level remained constant even in the presence of AP21967. The CBB-stained PVDF membrane is shown as the loading control.

Supplementary Figure S5

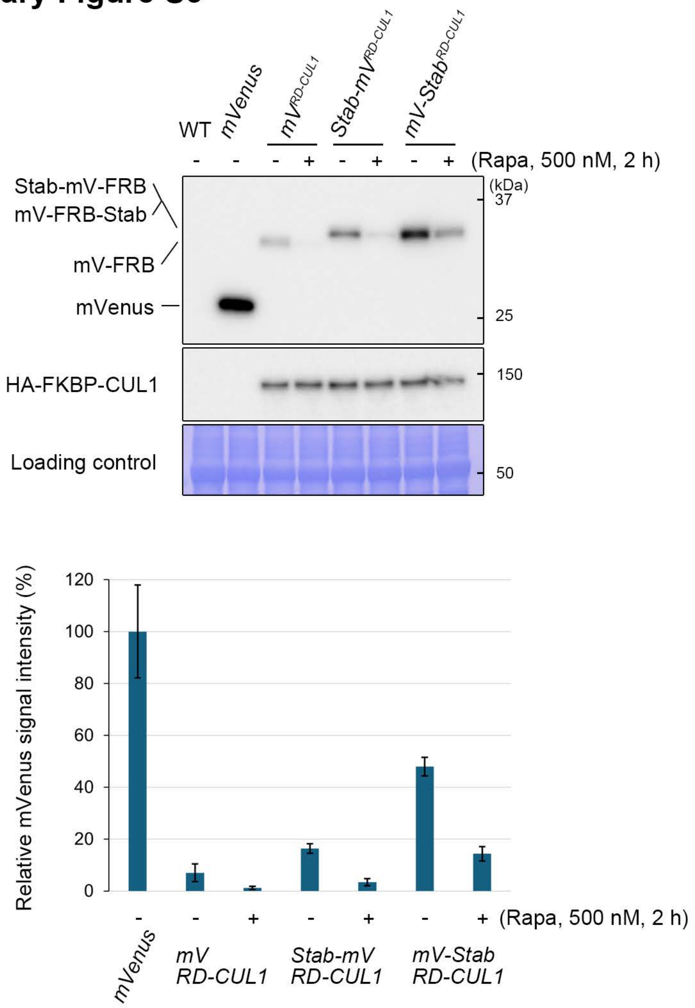

Supplementary Figure S5. The addition of a Stabilon tag improved the stability of mVenus-FRB.

A Stabilon tag was added to either N- (*Stab-mV*$^{RD-CUL1}$) or C-terminal (*mV-Stab*$^{RD-CUL1}$) of mVenus-FRB (*mV-FRB*) and expressed in the HA-FKBP-CUL1 expressing strain. Immunoblotting with the anti-GFP antibody comparing mVenus or mV-FRB protein level in WT, *mV*$^{RD-CUL1}$, *Stab-mV*$^{RD-CUL1}$, and *mV-Stab*$^{RD-CUL1}$ strains 2 h after the addition of DMSO (–) or 500 nM rapamycin (+). Also shown is an immunoblotting with the anti-HA antibody showing the level of HA-FKBP-CUL1. The CBB-stained PVDF membrane is shown as a loading control. The graph shows the mVenus or mV-FRB protein level. The signal intensities of the mVenus or mVenus-FRB bands on the immunoblot were quantified with ImageJ software, with the mVenus level in the mVenus expressing strain set to 100%. The values represent the mean of triplicate of the immunoblotting results. and the bars indicate the standard deviation. The same clones were analyzed three times for both the *mVenus* and *mV*$^{RD-CUL1}$ strains, whereas three independent transformed clones were analyzed for the *Stab-mV*$^{RD-CUL1}$ and *mV-Stab*$^{RD-CUL1}$ strains.
