# Supplementary material for: Development of a rapamycin-inducible protein-knockdown system in the unicellular red alga Cyanidioschyzon merolae
Source: Plant Physiol. 2024 Jun 4;196(1):77–94. doi: 10.1093/plphys/kiae316 (PMC11376382; doi:10.1093/plphys/kiae316)
Supplement: kiae316_Supplementary_Data [file pp.02005.2023-s05.pdf › pp.02005.2023-s05.pdf]

Plant Physiology®

Development a rapamycin-inducible protein knockdown system in the unicellular red alga Cyanidioschyzon merolae

Takayuki Fujiwara, Shunsuke Hirooka, Shota Yamashita, Fumi Yagisawa, and Shin-ya Miyagishima

Review Timeline:

Submission Date:	14-Dec-2023
Editorial Decision:	01-Feb-2024
Revision Received:	25-Apr-2024
Accepted:	14-May-2024

01-Feb-2024

Dr. Takayuki Fujiwara
Kokuritsu Idengaku Kenkyujo
Department of Gene Function and Phenomics
MIshima, Shizuoka
Japan

RE: Development a rapamycin-inducible protein knockdown system in the unicellular red alga Cyanidioschyzon merolae

Dear Dr. Fujiwara:

We have received reviews of your manuscript, which you may view at the end of this letter. On the basis of the advice received and my own evaluation, I believe that, pending revision, this article may ultimately be acceptable for publication in Plant Physiology.

The reviewers are supportive and provide clear guidance on important revisions to improve the manuscript. I would also like to note that claims of novelty/priority are generally not encouraged and draw your attention to the following papers that seem of relevance for this approach. Please cite/discuss them as appropriate, or if not of relevance then please note this in your response to the reviewers.
https://pubmed.ncbi.nlm.nih.gov/27208282/
https://pubmed.ncbi.nlm.nih.gov/28986906/

When you are ready to submit your revision, click the below link and find the red arrow under your Author Tasks heading for this revision submission.

If you want to visit this version of the manuscript, you may click the link below and then click on the folder that reads "Post Decision Manuscripts".

Link Not Available

When uploading the revised version of this article, please be sure to include as complete an answer as possible to the referees' criticisms during the first round of reviews in the "response to reviewers" section.

Plant Physiology considers a revision for up to 60 days after a manuscript has been provisionally accepted; limited extension of this timeframe is at the discretion of the Monitoring Editor. A reminder notice will be sent to you 2 weeks before the deadline, at which time you may choose to contact the editorial office to request an extension; if so, please provide a justification for your request. After the expiration of this deadline, the article would need to be submitted as a resubmission and would be handled as such.

Thank you very much for giving us an opportunity to review this work. I look forward to receiving the next version.

Sincerely,

Editor, Plant Physiology

Want to add this revision deadline to your calendar? Click below!

-- Reviewer comments:
Reviewer #1 (Comments for the Author):

In this study the authors developed a constitutive and a rapamycin-inducible system for targeted knock-down of proteins (mVenus and endogenous proteins) by fusing them with the FRB domain of human TOR kinase. Knockdown efficiency was

enhanced by fusing human FKBP to components of C. merolae's SCF complex. This is the first protein knockdown system reported for photosynthetic eukaryotes and it is particularly useful as it enables the study of proteins whose gene disruption would be lethal. Experimental conduction and documentation are mostly convincing and limitations of the method are discussed extensively. There is some inconsistency in the lines used/shown but overall this manuscript is a very valuable methodological paper.
Major comments
Fig. 1 et al. A caveat is the instability caused by FRB fusion even without Rapamycin addition, e.g for YFP and DRP5B. This is extensively discussed and excellent suggestions made to overcome this issue. However, the constructs/lines, e.g. addition of the STABILON tag, are not generated which would have been certainly a very valuable addition.
Fig. 5 and 6. It would be good to show some more cells after treatment. Fig. 5: the DRP5B strain that has been treated with DMSO or Rapamycin for 24 h should be shown. Fig. 6: Cells are not shown at all and this should be included.
Fig. 2E and S1B: It is clear that the medians plotted in 2E refer to the histograms of S1C. However, error bars and a statistical comparison to Fig 2E should be provided.
L. 295: I find it a bit unexpected that DRP5B is selected as endogenous target protein as it would have been more obvious to again test RB as in the constitutive system and test if the protein level (which had been 40 %) can be further decreased by rapamycin treatment.

Minor comments
L.2: Development OF a rapamycin-inducible ...
L.43: maybe here the authors could point out that this system enables to study the functions of proteins whose loss would have been lethal if it had already occurred at gene disruption level (as they do later in L. 83-85 but this information is so relevant that I would put it in the abstract)
L.81: C. merolae's
L. 85; maybe also point out that this is a method that can proof that the failure of generating a loss-of-function mutation is due to the loss of a gene being actually lethal instead of due to a technical error in the transformation process
L.123: reference?
L.129: THEY are referred to ....
L.139: "...indicate AN enhanced degradation efficiency of the FRB* tag over the FRB tag, and THAT the..."
Fig. 2C and E: Even with the triple tags the reduction only goes down to around 40 % and it is not clear if this is sufficient. The authors determine the average cell volumina of the mutants. If they would be compared to the cell volumina of the RB knock out cells that were produced in a different study (L. 158) it could be discussed if the 40 % reduction is sufficient to have a significant physiological effect.
L.278: maybe a sentence that aims to explain the results of the RNA-seq might be useful
Fig. 4C and D: typo in legends: relative level of mVenus-FRB
L. 303: It is not clear why HA-FKBP-SKP1 is used here, because in the previous section it was shown that CUL1 was more efficient.
L.335: If still possible, it might be helpful to measure the intensities of mV-FL-FRB-DRP5B protein level in the presence and absence of Rapamycin at each time point, and either plot them or add them as numbers as in Fig. 2C.
L.382: same comment as for L.335.
L.468: Fusing FRB to CUL1 or SKP1 might decrease their protein levels due to the destabilizing effect of FRB which might in turn affect the protein levels of all targets of CUL1 or SKP1. This would mean that to be sure that the effect is only from knockdown of the POI, the levels of all target proteins would need to be determined.
L.567: The SDs in Fig. 1C indicate that the immunoblotting was repeated several times but the methods don't say how many times.
L.940: Maybe it would be helpful for the reader if it was indicated here that the effect of the three nonsense mutations increases the destabilizing effect of FRB.
Fig. S3: growth of the cells has been monitored for only two days, which is rather short as stable lines are generated. Additional timepoints should be added.

Reviewer #2 (Comments for the Author):

Fujiwara et al report a constitutive and an inducible protein knockdown system for the red alga C. merolae. This is the first report (but see below, specific comments) of a protein knockdown system in a photosynthetic eukaryote. For the constitutive system, the authors fuse an orthogonal human FRB domain of the TOR kinase to the target protein, which destabilizes the target. For the inducible system, FRB is fused to the target and FKBP is fused to an endogenous adapter protein of the UBQ-proteasome system. Addition of rapamycin leads to dimerization of the FRB-tag with the FKBP-adapter with the FKBP adaptor, which is then ubiquitinated and degraded by the proteasome. The newly designed protein knockdown systems were then applied to the marker protein mVenus and several endogenous proteins, followed by phenotyping of the transformants.
The availability of an inducible protein knockdown system in C. merolae is particularly important because RNAi cannot be applied in this model alga, due to the lack of RNAi pathway components in its genome.

The constitutive system was first tested with mVenus. mVenus was fused to different variants of the FRB tag (single or triple

FRB or FRB*) from a chromosomal locus, and the mVenus signal in the transformants was assessed by fluorescence microscopy and immunoblotting. The strongest knockdown effects were observed with triple FRB / FRB*, with no significant differences between the two FRB versions.

Next, the FRB tag was fused to retinoblastoma protein (RB), an inhibitor of the G1/S transition in the cell cycle. The constructs were expressed from the chromosomal RB locus (inserted into the authentic locus by homologous recombination, very elegant work). A 60% reduction in RB protein was detected by immunoblots and a reduction in cell size was observed. The latter was expected from previous results with mutant RB.

The conditional was first prototyped with the mVenus reporter and then applied to the knockdown of two endogenous proteins, dynamin-related chloroplast division protein 5B (DRP5B) and the nuclear-localized transcription-factor E2F. Knockdown lines were phenotyped for chloroplast division (DRP5B) or for growth rate and transcriptome changes (EF2). There is no need to recapitulate the results here, suffice it to say that the observed phenotypes are consistent with expectations from previous work and thus nicely demonstrate the utility of the newly developed system.

It could be argued that rapamycin or expression of human FRB or FKBP affects growth or other aspects in C. merolae. This was tested by transcriptomic analysis. No effects of rapamycin on the transcriptome were observed in the wild type. In mVRD-SKP1 30 upregulated genes and 16 downregulated genes were observed. The implications of this finding are properly discussed and possible solutions (derivatives of rapamycin) are mentioned. Testing these is beyond the scope of the current study.

Overall, this is an excellent paper. The approach presented is directly applicable to C. merolae and will inspire modified versions that can be applied to land plants. I have only a few minor specific comments:

i) The constitutive knockdown of mVenus lacks an internal control. The amount of mVenus protein should be reported relative to the amount of a non-tagged protein on the same construct. For example, if antibodies are available, this could be the URA3 marker.
ii) The concept of constitutive FRB fusion and rapamycin-inducible FRB-FKBP adapter dimerization needs to be better explained. It remained unclear what is the difference (if any) between mVenus-FRB in Figure 1 and mVenus-FRB in Figure 3 and why the mVenus-FRB in the inducible system is not destabilized in the absence of rapamycin.
iii) Report the number of independent biological replicates not only in the figure legends but also in the Materials and Methods section.
iv) Fig. 1: Quantification of immunoblots: the figure legend states that triplicates of immunoblot results were quantified; are these from independent experiments or are they technical replicates (triplicate Westerns from the same sample)?)?
v) Fig 2: were the immunoblots been replicated?
vi) Figure 2E: no statistics shown
vii) Make all plasmid and construct data available as supplementary material (currently it says: "available on request", which is not in line with open science.

Dear Dr. Monitoring Editor of *Plant Physiology*,

We thank you, the handling editor and the reviewers for very helpful and important comments to improve our manuscript (ID PP2023-RA-02005). We have considered all of the comments carefully and made the following revisions, which are highlighted in the uploaded text file. The ed-itor's and reviewers' comments were copied and underlined, and we have described our revisions directly beneath each comment.

[Editor's comment]

We have received reviews of your manuscript, which you may view at the end of this letter. On the basis of the advice received and my own evaluation, I believe that, pending revision, this article may ultimately be acceptable for publication in Plant Physiology.

The reviewers are supportive and provide clear guidance on important revisions to improve the manuscript. I would also like to note that claims of novelty/priority are generally not encouraged and draw your attention to the following papers that seem of relevance for this approach. Please cite/discuss them as appropriate, or if not of relevance then please note this in your response to the reviewers.
https://pubmed.ncbi.nlm.nih.gov/27208282/
https://pubmed.ncbi.nlm.nih.gov/28986906/

Thank you for your evaluation and helpful comments on our manuscript. We have cited the papers you provided and revised the discussion based on them (lines 526–529, 537–540). In addition, while revising this manuscript, another paper was published. This paper developed a protein knockdown system utilizing the Salmonella-secreted protein H1 (SspH1) and the HR1b domain of protein kinase N1 (PKN1) in plants (Huang and Rojas-Pierce, 2024). This paper has also been cited, and its contents have been described and discussed (529–540).

[Reviewer #1's comments]

In this study the authors developed a constitutive and a rapamycin-inducible system for targeted knock-down of proteins (mVenus and endogenous proteins) by fusing them with the FRB domain of human TOR kinase. Knockdown efficiency was enhanced by fusing human FKBP to components of C. merolae's SCF complex. This is the first protein knockdown system reported for photosynthetic eukaryotes and it is particularly useful as it enables the study of proteins

whose gene disruption would be lethal. Experimental conduction and documentation are mostly convincing and limitations of the method are discussed extensively. There is some inconsistency in the lines used/shown but overall this manuscript is a very valuable methodological paper.

Thank you very much for your evaluation on our manuscript. We have revised the manuscript according to your suggestions as describe below.

Major comments

[1-1] Fig. 1 et al. A caveat is the instability caused by FRB fusion even without Rapamycin addition, e.g for YFP and DRP5B. This is extensively discussed and excellent suggestions made to overcome this issue. However, the constructs/lines, e.g. addition of the STABILON tag, are not generated which would have been certainly a very valuable addition.

We thank you for your suggestion. We have tested the effect of addition of Stabilon tag to either the N or C terminals of mVenus-FRB as suggested. The results are shown in Fig. S5 and the results have been explained and discussed in the text (lines, 318–344 and 549–550). In brief, the addition of the Stabilon tag improved the stability of mVenus-FRB.

[1-2] Fig. 5 and 6. It would be good to show some more cells after treatment. Fig. 5: the DRP5B strain that has been treated with DMSO or Rapamycin for 24 h should be shown.

[1-3] Fig. 6: Cells are not shown at all and this should be included.

Thank you for your suggestion. In Fig. 5, we have added the image of cells treated with DMSO or Rapamycin at hour 24 the in Fig. 5C and revised the legend (lines 1206, 1211). In Fig. 6, we have added the images of the cells after the addition of DMSO or rapamycin (Fig. 6C) and compared the frequency of dividing (S- and M-phase) cells (Fig. 6C). In addition, we performed immunostaining with the anti-PCNA (an S-phase marker) antibody to quantify S-phase cells in the DMSO- and rapamycin-treated cultures (Fig. 6D), and revised the text (lines 430–450 in the results; 676-686 in Materials and Methods; 1236–1248 in the figure legends 6C and D).

[1-4] Fig. 2E and S1B: It is clear that the medians plotted in 2E refer to the histograms of S1C. However, error bars and a statistical comparison to Fig 2E should be provided.

Thank you for pointing it out. We have revised the graph in Fig 2E, showing the box plot comparing the cell volume of WT, *HA-RB*, the three types of *HA-FRB-RB*, and *RB-KO* strains. We also added the information of statistical comparison.

[1-5] L. 295: I find it a bit unexpected that DRP5B is selected as endogenous target protein as it would have been more obvious to again test RB as in the constitutive system and test if the protein level (which had been 40 %) can be further decreased by rapamycin treatment.

We appreciate your suggestion. Implementing rapamycin-inducible knockdown of RB, as you suggested, would indeed provide a concise and coherent storyline. However, we decided not to further investigate RB due to its non-essential role in cell growth and survival, as well as the sufficient analysis of its phenotype. Additionally, our parallel experimentation initially prioritized DRP5B, aiming to demonstrate the versatility of our approach in studying various protein types.

Minor comments

[1-6] L.2: Development OF a rapamycin-inducible ...

Thank you for pointing it out. We have fixed the error (line 2).

[1-7] L.43: maybe here the authors could point out that this system enables to study the functions of proteins whose loss would have been lethal if it had already occurred at gene disruption level (as they do later in L. 83-85 but this information is so relevant that I would put it in the abstract)

We thank you for pointing it out. We have added this point to the abstract according to your suggestion (lines 41–46).

[1-8] L.81: C. merolae's

Thank you for pointing it out. We have fixed the error (line 84).

[1-9] L. 85; maybe also point out that this is a method that can proof that the failure of generating a loss-of-function mutation is due to the loss of a gene being actually lethal instead of due to a technical error in the transformation process.

Thank you for pointing it out. We have added this point to the text (lines 104–107).

[1-10] L.123: reference?

Thank you for pointing it out. We have added a reference (lines 135–136; Stankunas et al., 2003).

[1-11] L.129: THEY are referred to ....

Thank you for pointing it out. We have fixed the error (line 141).

[1-12] L.139: "...indicate AN enhanced degradation efficiency of the FRB* tag over the FRB tag, and THAT the..."

Thank you for pointing it out. We have fixed the error (lines 152–153).

[1-13] Fig. 2C and E: Even with the triple tags the reduction only goes down to around 40 % and it is not clear if this is sufficient. The authors determine the average cell volume in all of the mutants. If they would be compared to the cell volume of the RB knock out cells that were produced in a different study (L. 158) it could be discussed if the 40 % reduction is sufficient to have a significant physiological effect.

Thank you for your suggestion. We have added the results of *RB-KO* (Fig. 2D and E; Fig. S1), and revised the text (lines 180–182, 195–197).

[1-14] L.278: maybe a sentence that aims to explain the results of the RNA-seq might be useful.

Thank you for your suggestion. We have added the explanation of the results to the text (lines 302–304).

[1-15] Fig. 4C and D: typo in legends: relative level of mVenus-FRB

Thank you for pointing it out. We have fixed the errors (lines 1153 and 1160).

[1-16] L. 303: It is not clear why HA-FKBP-SKP1 is used here, because in the previous section it was shown that CUL1 was more efficient.

Your suggestion is indeed correct. Using the HA-FKBP-CUL1 system, as you proposed, would provide a concise and coherent storyline. However, we were concurrently conducting experiments presented in the manuscript. Results for DRP5B with HA-FKBP-SKP1 were obtained first and demonstrated a sufficiently interpretable phenotype. For these reasons, we did not pursue further experiments with DRP5B using the HA-FKBP-CUL1 system.

[1-17] L.335: If still possible, it might be helpful to measure the intensities of mV-FL-FRB-DRP5B protein level in the presence and absence of Rapamycin at each time point, and either plot them or add them as numbers as in Fig. 2C.

Thank you for pointing it out. We have measured the intensities of mV-FL-FRB-DRP5B protein level, added the results (Fig. 5B and C; mV-FL-FRB-DRP5B), and revised the figure legend 5B and C (lines 1185–1186 and 1195–1197).

[1-18] L.382: same comment as for L.335.

Thank you for pointing it out. We have revised Fig. 6B.

[1-19] L.468: Fusing FRB to CUL1 or SKP1 might decrease their protein levels due to the destabilizing effect of FRB which might in turn affect the protein levels of all targets of CUL1

or SKP1. This would mean that to be sure that the effect is only from knockdown of the POI, the levels of all target proteins would need to be determined.

Thank you for highlighting this important aspect. In this study, we expressed FKBP-SKP1 or FKBP-CUL1 from a chromosomal neutral locus rather than from the SKP1 or CUL1 locus. Therefore, FKBP-SKP1 or FKBP-CUL1 was expressed in addition to the endogenous SKP1 or CUL1. As you mentioned, fusing FRB to CUL1 or SKP1 likely reduces the level of the additionally expressed CUL1 or SKP1 but does not affect the level of endogenous SKP1 or CUL1. Thus, the expression of FRB-CUL1 or FRB-SKP1 is unlikely to affect the levels of target proteins of CUL1 or SKP1. To avoid potential misinterpretation, we removed "endogenous," which describes the SCF complex.

[1-20] L.567: The SDs in Fig. 1C indicate that the immunoblotting was repeated several times but the methods don't say how many times.

Thank you for pointing it out. We have added the information to the materials and methods (lines 649–664).

[1-21] L.940: Maybe it would be helpful for the reader if it was indicated here that the effect of the three nonsense mutations increases the destabilizing effect of FRB.

Thank you for pointing it out. We have added the information to the legend of Fig. 1B (lines 1060–1061).

[1-22] Fig. S3: growth of the cells has been monitored for only two days, which is rather short as stable lines are generated. Additional timepoints should be added.

Thank you for your suggestion. We have monitored the cells for 14 days and shown the results in Fig. S3A and revised the text (line 293)

[Reviewer #2’s comments]

Fujiwara et al report a constitutive and an inducible protein knockdown system for the red alga C. merolae. This is the first report (but see below, specific comments) of a protein knockdown system in a photosynthetic eukaryote. For the constitutive system, the authors fuse an orthogonal human FRB domain of the TOR kinase to the target protein, which destabilizes the target. For the inducible system, FRB is fused to the target and FKBP is fused to an endogenous adapter protein of the UBQ-proteasome system. Addition of rapamycin leads to dimerization of the FRB-tag with the FKBP-adapter with the FKBP adaptor, which is then ubiquitinated and degraded by the proteasome. The newly designed protein knockdown systems were then applied to the marker protein mVenus and several endogenous proteins, followed by phenotyping of the transformants.
The availability of an inducible protein knockdown system in C. merolae is particularly important because RNAi cannot be applied in this model alga, due to the lack of RNAi pathway components in its genome.

The constitutive system was first tested with mVenus. mVenus was fused to different variants of the FRB tag (single or triple FRB or FRB*) from a chromosomal locus, and the mVenus signal in the transformants was assessed by fluorescence microscopy and immunoblotting. The strongest knockdown effects were observed with triple FRB / FRB*, with no significant differences between the two FRB versions.

Next, the FRB tag was fused to retinoblastoma protein (RB), an inhibitor of the G1/S transition in the cell cycle. The constructs were expressed from the chromosomal RB locus (inserted into the authentic locus by homologous recombination, very elegant work). A 60% reduction in RB protein was detected by immunoblots and a reduction in cell size was observed. The latter was expected from previous results with mutant RB.

The conditional was first prototyped with the mVenus reporter and then applied to the knockdown of two endogenous proteins, dynamin-related chloroplast division protein 5B (DRP5B) and the nuclear-localized transcription-factor E2F. Knockdown lines were phenotyped for chloroplast division (DRP5B) or for growth rate and transcriptome changes (EF2). There is no need to recapitulate the results here, suffice it to say that the observed phenotypes are consistent with expectations from previous work and thus nicely demonstrate the utility of the newly developed system.

It could be argued that rapamycin or expression of human FRB or FKBP affects growth or other aspects in C. merolae. This was tested by transcriptomic analysis. No effects of rapamycin on

the transcriptome were observed in the wild type. In mVRD-SKP1 30 upregulated genes and 16 downregulated genes were observed. The implications of this finding are properly discussed and possible solutions (derivatives of rapamycin) are mentioned. Testing these is beyond the scope of the current study.

Overall, this is an excellent paper. The approach presented is directly applicable to C. merolae and will inspire modified versions that can be applied to land plants. I have only a few minor specific comments:

Thank you very much for your evaluation on our manuscript. We have revised the manuscript according to your suggestions as described below.

[2-1] The constitutive knockdown of mVenus lacks an internal control. The amount of mVenus protein should be reported relative to the amount of a non-tagged protein on the same construct. For example, if antibodies are available, this could be the URA3 marker.

Thank you for your suggestion. We have added result of immunoblotting using anti-eEF-2 (eukaryotic translation elongation factor 2) as an internal control (Fig. 1C) and the information on the antibody to the materials and methods section (line 640) because we did not have an anti-URA antibody. In addition, the CBB-stained PVDF membranes represent the different area of the same membrane used for immunoblotting of mVenus and mVenus-FRB.

[2-2] The concept of constitutive FRB fusion and rapamycin-inducible FRB-FKBP adapter dimerization needs to be better explained. It remained unclear what is the difference (if any) between mVenus-FRB in Figure 1 and mVenus-FRB in Figure 3 and why the mVenus-FRB in the inducible system is not destabilized in the absence of rapamycin.

Thank you for your suggestion. We have revised the manuscript (lines 108–121 in the introduction; lines 208–211, 227–231 in the results).

[2-3] Report the number of independent biological replicates not only in the figure legends but also in the Materials and Methods section.

We thank you for pointing out it. We have added the information to the materials and methods (lines 649–664)

[2-4] Fig. 1: Quantification of immunoblots: the figure legend states that triplicates of immunoblot results were quantified; are these from independent experiments or are they technical replicates (triplicate Westerns from the same sample)?

We thank you for pointing out it. In Fig. 1 they were technical replicates. We have revised the figure legend (line 1074) and added the information to the materials and methods (lines 649–664).

[2-5] Fig 2: were the immunoblots been replicated?

The data were obtained through single immunoblotting. We have added the information to the materials and methods (lines 649–664).

[2-6] Figure 2E: no statistics shown.

Thank you for pointing it out. We have revised Fig. 2E.

[2-7] Make all plasmid and construct data available as supplementary material (currently it says: "available on request", which is not in line with open science.

Thank you for pointing it out. We have added the information on the DNA sequences of the constructs used in this study in Table S2.

We believe that our manuscript has been improved, thanks to the editor and reviewers, and hope it is now acceptable for publication. I look forward to your decision.

Yours sincerely,

Takayuki Fujiwara
Shin-ya Miyagishima

14-May-2024
Dr. Takayuki Fujiwara
Kokuritsu Idengaku Kenkyujo
Department of Gene Function and Phenomics
MIshima, Shizuoka
Japan

MSID: PP2023-RA-02005R1
MS TITLE: Development a rapamycin-inducible protein knockdown system in the unicellular red alga Cyanidioschyzon merolae

Dear Dr. Takayuki Fujiwara:

I am pleased to inform you that your revised manuscript "Development a rapamycin-inducible protein knockdown system in the unicellular red alga Cyanidioschyzon merolae" has been accepted for publication in Plant Physiology. Your manuscript will be checked for consistency with journal formatting and image standards by one of our Science Editors, who will contact you shortly. They may have a number of queries and suggestions for you. Please keep in mind that they work to increase the online visibility of your article and its attraction for readers from the widest of backgrounds. You will have the opportunity to review any revisions, and I hope you will work constructively with them to get the most out of your research. You will also have the opportunity to review all changes when you receive the author proofs for your article.

Please ensure the minor typos mentioned by R#1 are corrected during proofing.

Once validated for publication, your article will be sent to a Science Editor for a final review of your manuscript and source files. After your final files are uploaded, your article will appear in the next available issue and the online Preview version of your article will be posted after your signed Copyright and Checklist forms and final source files are received.

Please be sure to include the following in your manuscript file:
Harvard style citations
List of all author contributions and funding information at the beginning of manuscript after the titles and author list.
List all supplemental materials associated with the article, with titles, at the end of the ms, before the references and acknowledgments section.
Complete Figure Legends for all figures appearing in the article listed before the Literature Cited.

Thank you and congratulations on your Plant Physiology paper!

Sincerely,
Editor, Plant Physiology

=====
FIRST AUTHOR PROFILES
=====

We recognize the hard work that goes into being First Author of a Plant Physiology paper by publishing a "First Author Feature" for each First Author alongside your paper.

If the first author(s) would like to submit information for a feature on Facebook and Twitter, fill out the form by clicking on the link below:

Plant Physiology First Author Feature Form: https://forms.office.com/r/ZBCGZfhm8F
If you cannot access the form through the link above, please contact Rachel at rbelsky@aspb.org.

To upload an image of yourself to accompany your profile, please put your photo in the correct journal folder at the link below. You must label your image: Author First Name Last Name Article Title.

First Author Profile Image Dropbox: https://www.dropbox.com/scl/fo/xz029p7qjr2jml2rp2kmq/h?dl=0&rlkey=6fkxcaqmxx35ap152b2osy1fz

=====
IMPORTANT REMINDER: PEER REVIEW REPORTS
=====

If you opted to publish a peer review report along with your article during the original submission process, it will be prepared by the editorial staff and publicly posted with your manuscript, inside the zip file that contains any other supplemental material. As a reminder, the peer review report is a public record of all comments from editors and reviewers, as well as your prior responses, as you received them in the decision letters for each draft of your manuscript. If you agreed to publish this report and have changed your mind, or are not sure if you selected this option, please contact the editorial office as soon as possible before signing the license agreement from our publisher.

-- Reviewer comments:
Reviewer #1 (Comments for the Author):

I have only few minor comments left, which are rather typing errors

Line 125: a word after "nuclear" is missing
131: C-terminal region / C-terminus; the double-mutant
132: nuclear surface area; compared
135: N-terminal region / N-terminus

Reviewer #2 (Comments for the Author):

All of my comments on the original version have been satisfactorily addressed in revision.
